# Development and Validation of a Joint Attention–Based Deep Learning System for Detection and Symptom Severity Assessment of Autism Spectrum Disorder

**DOI:** 10.1001/jamanetworkopen.2023.15174

**Published:** 2023-05-25

**Authors:** Chanyoung Ko, Jae-Hyun Lim, JaeSeong Hong, Soon-Beom Hong, Yu Rang Park

**Affiliations:** 1 Department of Biomedical Systems Informatics, Yonsei University College of Medicine, Seoul, South Korea; 2 LumanLab Inc, Seoul, South Korea; 3 Division of Child and Adolescent Psychiatry, Department of Psychiatry, Seoul National University College of Medicine, Seoul, South Korea

## Abstract

**Importance:**

Joint attention, composed of complex behaviors, is an early-emerging social function that is deficient in children with autism spectrum disorder (ASD). Currently, no methods are available for objectively quantifying joint attention.

**Objective:**

To train deep learning (DL) models to distinguish ASD from typical development (TD) and to differentiate ASD symptom severities using video data of joint attention behaviors.

**Design, Setting, and Participants:**

In this diagnostic study, joint attention tasks were administered to children with and without ASD, and video data were collected from multiple institutions from August 5, 2021, to July 18, 2022. Of 110 children, 95 (86.4%) completed study measures. Enrollment criteria were 24 to 72 months of age and ability to sit with no history of visual or auditory deficits.

**Exposures:**

Children were screened using the Childhood Autism Rating Scale. Forty-five children were diagnosed with ASD. Three types of joint attention were assessed using a specific protocol.

**Main Outcomes and Measures:**

Correctly distinguishing ASD from TD and different levels of ASD symptom severity using the DL model area under the receiver operating characteristic curve (AUROC), accuracy, precision, and recall.

**Results:**

The analytical population consisted of 45 children with ASD (mean [SD] age, 48.0 [13.4] months; 24 [53.3%] boys) vs 50 with TD (mean [SD] age, 47.9 [12.5] months; 27 [54.0%] boys). The DL ASD vs TD models showed good predictive performance for initiation of joint attention (IJA) (AUROC, 99.6% [95% CI, 99.4%-99.7%]; accuracy, 97.6% [95% CI, 97.1%-98.1%]; precision, 95.5% [95% CI, 94.4%-96.5%]; and recall, 99.2% [95% CI, 98.7%-99.6%]), low-level response to joint attention (RJA) (AUROC, 99.8% [95% CI, 99.6%-99.9%]; accuracy, 98.8% [95% CI, 98.4%-99.2%]; precision, 98.9% [95% CI, 98.3%-99.4%]; and recall, 99.1% [95% CI, 98.6%-99.5%]), and high-level RJA (AUROC, 99.5% [95% CI, 99.2%-99.8%]; accuracy, 98.4% [95% CI, 97.9%-98.9%]; precision, 98.8% [95% CI, 98.2%-99.4%]; and recall, 98.6% [95% CI, 97.9%-99.2%]). The DL-based ASD symptom severity models showed reasonable predictive performance for IJA (AUROC, 90.3% [95% CI, 88.8%-91.8%]; accuracy, 84.8% [95% CI, 82.3%-87.2%]; precision, 76.2% [95% CI, 72.9%-79.6%]; and recall, 84.8% [95% CI, 82.3%-87.2%]), low-level RJA (AUROC, 84.4% [95% CI, 82.0%-86.7%]; accuracy, 78.4% [95% CI, 75.0%-81.7%]; precision, 74.7% [95% CI, 70.4%-78.8%]; and recall, 78.4% [95% CI, 75.0%-81.7%]), and high-level RJA (AUROC, 84.2% [95% CI, 81.8%-86.6%]; accuracy, 81.0% [95% CI, 77.3%-84.4%]; precision, 68.6% [95% CI, 63.8%-73.6%]; and recall, 81.0% [95% CI, 77.3%-84.4%]).

**Conclusions and Relevance:**

In this diagnostic study, DL models for identifying ASD and differentiating levels of ASD symptom severity were developed and the premises for DL-based predictions were visualized. The findings suggest that this method may allow digital measurement of joint attention; however, follow-up studies are necessary for further validation.

## Key Points

**Question:**

Can joint attention be quantified for detecting autism spectrum disorder (ASD) and assessing ASD symptom severities?

**Findings:**

In this diagnostic study of 45 children with ASD and 50 with typical development, a deep learning system trained on videos acquired using a joint attention–eliciting protocol for classifying ASD vs typical development and predicting ASD symptom severity showed high predictive performance. This new artificial intelligence–assisted approach based predictions on participants’ behavioral responses triggered by social cues.

**Meaning:**

These findings suggest that this method may allow scalable, digitalized measurement of joint attention, enabling deep learning–based analysis and modeling to facilitate development of automated detection and symptom severity assessment tools for individuals with social deficiencies.

## Teaser

This diagnostic study demonstrates a joint attention–based deep learning model that distinguishes children with autism spectrum disorder from those with typical development and differentiated autism spectrum disorder symptom severity levels.

## Introduction

Attending to other people and sharing an attentional focus on objects or events with other individuals facilitates learning to socialize.^1^ This is termed *joint attention*, and despite interindividual differences in joint attention, this ability is observed as early as 6 months in a typically developing infant.^2,3^ Autism spectrum disorder (ASD) is a neurodevelopmental disorder characterized by abnormal patterns of social interaction and communication.^4^ Infants with this condition, but not those with typical development (TD), appear to lack joint attention.^5^ Thus, this difference has interested researchers as a means of diagnosis, a prognostic indicator, and a potential intervention target for individuals with ASD.^6^ Although validated manuals for observing joint attention exist, for example, the Early Social Communication Scales (ESCS),^7^ they are labor-intensive and difficult to implement without trained clinicians and the proper experimental setup.

Recently, machine learning and deep learning (DL) artificial intelligence (AI) models using simple behavioral video data to detect ASD have gained momentum, not only to characterize children with autism objectively but also to develop scalable screening or assistive diagnostic tools for ASD.^8,9,10,11^ Previous efforts to build ASD-detection AI models showed promising results. However, due to a lack of automation, the requirements for specialized and expensive equipment, calibration, trained personnel, dependence on human rating of autistic behaviors, and low precision and recall, they are unsuitable for the development of ASD detection tools.^9,10,11^ The low precision and recall scores of these models^9,10,11^ may be due to a lack of targeted behavioral biomarkers that can readily discriminate ASD from TD. Another valid cause may be the absence of methods for quantitatively measuring the complex hallmark behaviors of ASD. Thus, there is a need for objective measurement of clinically validated autism-related behaviors, such as joint attention, which may be implemented in a screening tool as well as in an objective diagnostic tool for clinicians who have had to rely on subjective assessment scores in diagnosing ASD to date.

We developed a digitalized method for joint attention assessment that required a new protocol for specific task administration guidelines to elicit 3 types of joint attention mentioned in the ESCS^7^ for video recording of task-related behaviors. The collected video data were then used as input for training a DL model to identify ASD and assess ASD symptom severity. Preliminary results have been obtained previously with this approach in a small sample.^12^ In the present study, we assessed whether the joint attention–based DL model could distinguish children with ASD from those with TD and differentiate ASD symptom severity levels based on joint attention behaviors ascertained from input video data using explainable AI techniques.

## Methods

### Study Design and Setting

This prospective diagnostic study involved children aged 24 to 72 months from multiple sites in South Korea. Children with ASD were recruited from a single institution—Seoul National University Hospital (SNUH) Child Psychiatry Outpatient Clinic—where individuals at high risk of ASD were referred from throughout the country, while individuals with TD and no history of developmental delay or psychiatric condition per parent report were recruited from various day care centers across South Korea. Participants were from Seoul (25%), metropolitan cities (25%), or self-governing provinces (50%). All caregiver-reported ethnicity, in accordance with the National Institutes of Health categories, was Korean. Caregivers provided written informed consent. The study was approved by Yonsei University Health System Institutional Review Board and followed the Standards for Reporting of Diagnostic Accuracy Studies (STARD) reporting guideline.

### Participants

Conservative inclusion criteria for ASD (clinical diagnosis by a child psychiatrist and scores above the cutoff on the criterion standard diagnostic tool) and TD (scores below the cutoff on a screening tool) were used to select individuals belonging to clinically distinct groups to ensure development of an accurate and precise ASD vs TD classification model. Detailed enrollment process is described in eFigure 1 in Supplement 1, and sociodemographic and clinical measures are presented in the Table.

**Table.** Participant Characteristics^a^

Characteristic	Participant group		*P* value
	ASD (n = 45)	TD (n = 50)	
Recruitment	University hospital	Day care centers	>.99
Sex, No. (%)			
Boys	24 (53.3)	27 (54.0)	>.99
Girls	21 (46.7)	23 (46.0)	
Age, mo			
Mean (SD)	48.0 (13.4)	47.9 (12.5)	.99
Median (range)	52.0 (24.0-68.0)	48.5 (25.0-72.0)	
Toddler or preschool age, No. (%)			
<48 mo	21 (46.7)	21 (42.0)	.80
≥48 mo	24 (53.3)	29 (58.0)	
Best estimate IQ by age^b^			
<48 mo			
Mean (SD)	60.1 (15.4)	104 (19.1)	<.001
Median (range)	60.0 (40.0-95.0)	111 (55.0-125.0)	
≥48 mo			
Mean (SD)	55.1 (20.8)	105 (14.5)	<.001
Median (range)	42.0 (40.0-119.0)	105 (56.0-140.0)	
Verbal IQ by age^b^			
<48 mo			
Mean (SD)	61.5 (15.4)	103 (20.0)	<.001
Median (range)	55.0 (46.0-101.0)	106 (46.0-129.0)	
≥48 mo			
Mean (SD)	58.8 (20.8)	103 (18.3)	<.001
Median (range)	45.0 (45.0-127.0)	105 (62.0-142.0)	
K-CARS-2 by age^c^			
<48 mo			
Mean (SD)	31.3 (5.4)	16.0 (2.9)	<.001
Median (range)	31.5 (18.5-40.0)	15.0 (15.0-25.5)	
≥48 mo			
Mean (SD)	31.2 (6.1)	15.1 (0.3)	<.001
Median (range)	31.3 (32.0-43.0)	15.0 (15.0-16.0)	
K-ADOS-2 CSS^c^			
Mean (SD)	6.07 (1.6)	NA	NA
Median (range)	6.00 (3.0-10.0)	NA	
SA CSS			
Mean (SD)	6.62 (1.8)	NA	NA
Median (range)	6.00 (3.0-10.0)	NA	
RRB CSS			
Mean (SD)	6.16 (2.2)	NA	NA
Median (range)	7.00 (1.0-9.0)	NA	
K-ADOS-2 module, No. (%)^d^			
T	8 (17.8)	NA	NA
1	28 (62.2)	NA	
2	9 (20.0)	NA	

Abbreviations: ASD, autism spectrum disorder; CSS, calibrated severity score; K-ADOS-2, Korean Autism Diagnostic Observation Schedule II; K-CARS-2, Korean Childhood Autism Rating Scale II; NA, not applicable; RRB, restricted and repetitive behaviors; SA, social affect; T, toddler; TD, typical development.

^a^ The threshold for statistical significance was set at *P* < .05. χ^2^ Test was used to compare categorical variables (reported as No. [%] of participants); continuous variables are reported as mean (SD) or median (range).

^b^ Measured using Korean Wechsler Preschool and Primary Scale of Intelligence, Fourth Edition scores and Korean Bayley Scales of Infant and Toddler Development, Second Edition scores.

^c^ Measured using the K-ADOS-2 total severity calibrated scores.

^d^ Modules are designated T for toddler, 1 for individuals with preverbal or single-word language, and 2 for individuals with phrase speech.

### Measures

#### Screening Assessments

All caregivers completed the Korean Childhood Autism Rating Scale II (K-CARS-2)^13,14^ as part of screening. The K-CARS-2 consists of 15 questions about the presence or absence of autism symptoms, with a total score of 15 to 60. The K-CARS-2 has been also used to assess ASD symptom severity, where scores less than 30 represent non-ASD; 30 to 36, mild or moderate ASD; and 37 to 60, severe ASD.^14^

#### Diagnostic Assessments

Children whose K-CARS-2 scores revealed an ASD risk or whose caregiver expressed concern were referred to a child psychiatrist for a diagnostic evaluation at SNUH. The Korean Autism Diagnostic Observation Schedule II (K-ADOS-2)^15,16,17^ was administered to children who received a clinical diagnosis of ASD as part of routine evaluation.

#### Cognitive Functioning Assessment

Cognitive functioning was assessed using various measurements, depending on the child’s age and ability to attend to demanding cognitive tasks. We defined the best estimate IQ using the Korean Bayley Scales of Infant and Toddler Development, Second Edition,^18^ and Korean Wechsler Preschool and Primary Scale of Intelligence, Fourth Edition.^19^

#### Joint Attention Tasks and Video Data Acquisition

We designed a protocol for measuring and video recording 3 types of joint attention, adopting methods from the ESCS manual of Mundy et al^7^ and validated behavior extraction techniques.^20^ Children were individually tested in a quiet room. Each child was seated on a height-adjustable chair in front of a table.

There are 2 types of joint attention: initiation of joint attention (IJA) associated with the child’s motivation for social interaction, and response to joint attention (RJA), associated with the child’s responsiveness to a social cue.^21^ Response to joint attention may be classified as low and high level, referring to the child’s ability to maintain attention on objects pointed near them (low-level RJA) and far from them (high-level RJA).^22^ Video data were collected from 3 joint attention tasks designed to elicit IJA, low-level RJA, and high-level RJA behaviors. The procedures and video acquisition setup are described in eFigures 2 and 3 in Supplement 1. Video data were acquired in a single 10-minute session per participant. Tasks were filmed from a front-facing viewpoint using a digital camera (DSC-RX100 IV; Sony) with resolution of 1920 × 1080 and 30 frames/second. As a way of monitoring how engaged a participant was for each repeated trial of a given task, we devised a compliance score metric to compute compliance scores that could indirectly show the participants’ task performance and engagement level (eTable 1 in Supplement 1).

#### Development of a DL System for ASD Detection and Symptom Severity Assessment

We customized a DL classification system consisting of neural network architectures, that is, a convolutional neural network (CNN),^23^ long short-term memory,^24^ and attention mechanism^25^ as illustrated in eFigure 4 in Supplement 1. Size of the input data for the IJA-based DL system is 224 × 224 × 300 (30 frames/s × 10 seconds) and 224 × 224 × 150 (30 frames/s × 5 seconds) for the RJA-based DL system. We used 10-fold group-wise (by individual) cross-validation for development of ASD detection and ASD symptom severity assessment systems. Performances during training and validation are presented in eTable 2 in Supplement 1.

#### Interpretability of the DL System: Use of Class Activation Map, Attention Plot, and Cluster Map

The gradient-weighted class activation mapping (Grad-CAM) technique^26^ was used to produce visual explanations of how the system makes its prediction by superimposing a visualization layer at the end of the CNN model. This method uses the gradients of any target concept, which are accumulated in the last CNN layer, to generate a localization heatmap highlighting key areas in the image for predicting the concept.^26^ Redder areas suggest more significant features for model prediction. Long short-term memory was developed to better process sequential data^24^; hence, it was incorporated into our DL classification system to account for the time-dependent nature of video data. The addition of the attention mechanism^25^ enabled us to access and visualize the attention weights across each video sequence according to which frames or video time points contributed most to the model’s decision-making by plotting the attention weights across each video sequence. To visualize how the DL system distinguishes ASD from TD or distinguishes the different severities of ASD on a data set level and thus verify the consistency of our DL system’s decision-making, we drew cluster maps on the testing data set for each joint attention task. The technique used was agglomerative hierarchical clustering, which is characterized by clustering through iteration, where similar clusters merge with other clusters until *k* clusters are formed.^27^ This can be visualized via a dendrogram.^27^

### Statistical Analysis

We used means (SDs) and medians (ranges) to express continuous variables. The χ^2^ test was used to compare categorical variables. A 2-way mixed analysis of variance was used to explore 2-way interactions between the ASD vs TD effect and the number of repeated trials on the compliance score. The area under the receiver operating characteristic (AUROC), accuracy, recall, and precision were computed to evaluate the performance of the classification models. Statistical analyses and calculations of the validation measures were performed using Python, version 3.6.8, with SciPy, version 1.4.1,^28^ and Statsmodels 0.11.1 (Python Software).^29^ Cluster maps were drawn using Scikit-learn, version 0.23.2,^30^ Seaborn, version 0.11.0,^31^ and Matplotlib, version 3.3.1 (Python Software).^32^ Deep learning provided a classification score ranging from 0 to 1, and the lowest predicted probability value of the DL model’s output is greater than 0.5 for classifying ASD vs TD and the different ASD symptom severities.^11,33^ The threshold for statistical significance was set at 2-sided *P* < .05. We estimated CIs with the Hanley and McNeil method^34^ at 95% level.

## Results

### Participant Characteristics

Of the 110 children with screening data, a total of 95 (86.4%) were included for joint attention–based AI model training, 45 (47.4%) with ASD (mean [SD] age, 48.0 [13.4] months; 24 boys [53.3%] and 21 girls [46.7%]) and 50 (52.6%) with TD (mean [SD] age, 47.9 [12.5] months; 27 boys [54.0%] and 23 girls [46.0%]). Detailed descriptions of the 2 groups are shown in the Table.

### Task Compliance Through Repeated Trials by Diagnostic Group

Using our compliance scoring metric, we discovered that while the overall quality of joint attention behaviors differed between TD and ASD groups independently of trial or task type, each group showed similar within-group compliance (attentiveness) patterns for each joint attention task. While most children with ASD showed an incremental reduction in attentiveness after showing initial interest in the social cue as much as the children with TD for the IJA task, children with ASD showed a contrasting uninterest in the social cue presented at the beginning of RJA tasks. Detailed results of 2-way mixed analysis of variance and a visual representation of the task compliance results are shown in eFigure 5 in Supplement 1.

### Joint Attention–Based DL System for Prediction of ASD and ASD Symptom Severity 

**Figure 1.**
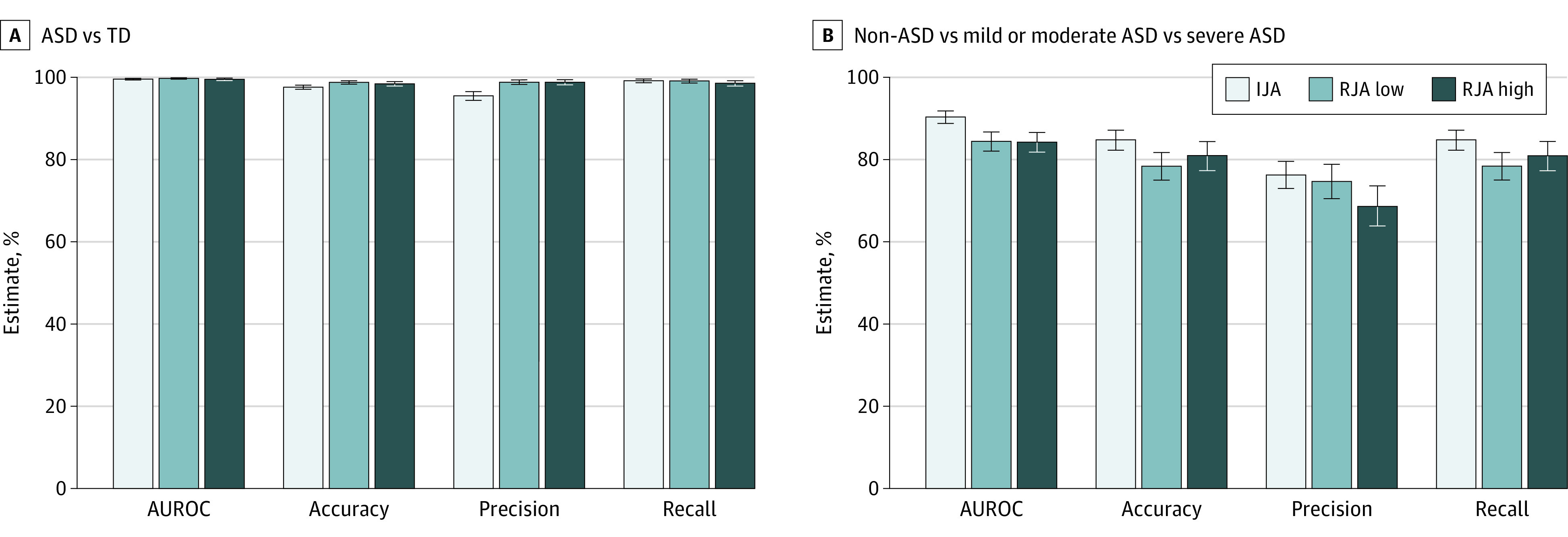
Performance of the Joint Attention–Based Deep Learning System Bar graphs of models for autism spectrum disorder (ASD) detection (A) and ASD symptom severity assessment (B). The models’ prediction of the area under the receiver operating characteristic curve (AUROC), accuracy, precision, and recall using initiation of joint attention and low- and high-level response to joint attention are shown for testing data sets. The mean and 95% CIs (error bars) were calculated from all ASD detection (A) and symptom severity assessment (B) models based on 10-fold cross-validation. IJA indicates initiation of joint attention; TD, typical development; RJA, response to joint attention.

While all DL models trained on any joint attention task showed promising classification performance in identifying ASD, the IJA-based ASD symptom severity prediction DL model showed superior performance compared with that of the models based on RJA tasks across all validation measures. The validation measures of the DL models are presented in Figure 1.

#### DL-Based ASD Prediction

The IJA-based model showed an AUROC of 99.6% (95% CI, 99.4%-99.7%), accuracy of 97.6% (95% CI, 97.1%-98.1%), precision of 95.5% (95% CI, 94.4%-96.5%), and recall of 99.2% (95% CI, 98.7%-99.6%). The low-level RJA–based model showed an AUROC of 99.8% (95% CI, 99.6%-99.9%), accuracy of 98.8% (95% CI, 98.4%-99.2%), precision of 98.9% (95% CI, 98.3%-99.4%), and recall of 99.1% (95% CI, 98.6%-99.5%). The high-level RJA–based model showed an AUROC of 99.5% (95% CI, 99.2%-99.8%), accuracy of 98.4% (95% CI, 97.9%-98.9%), precision of 98.8% (95% CI, 98.2%-99.4%), and recall of 98.6% (95% CI, 97.9%-99.2%).

#### DL-Based ASD Symptom Severity Prediction

The IJA-based model showed an AUROC of 90.3% (95% CI, 88.8%-91.8%), accuracy of 84.8% (95% CI, 82.3%-87.2%), precision of 76.2% (95% CI, 72.9%-79.6%), and recall of 84.8% (95% CI, 82.3%-87.2%). The low-level RJA–based model showed an AUROC of 84.4% (95% CI, 82.0%-86.7%), accuracy of 78.4% (95% CI, 75.0%-81.7%), precision of 74.7% (95% CI, 70.4%-78.8%), and recall of 78.4% (95% CI, 75.0%-81.7%). The high-level RJA–based model showed an AUROC of 84.2% (95% CI, 81.8%-86.6%), accuracy of 81.0% (95% CI, 77.3%-84.4%), precision of 68.6% (95% CI, 63.8%-73.6%), and recall of 81.0% (95% CI, 77.3%-84.4%). To explore whether age affects model performance, we performed additional analysis by age (<48 vs ≥48 months). Even after controlling for the effect of age, the IJA-based model performance was superior to that of the other task-based models. There was an improvement in high-level RJA–based model performance when trained on data sets of older children. The results of model performance by age group are presented in eFigure 6 in Supplement 1.

### Interpreting the DL System’s Classification Premises: Grad-CAM and Attention Plot

The Grad-CAM results for TD and ASD are shown in eFigure 7 in Supplement 1. Attention plots are shown in eFigure 8 in Supplement 1. Peaks at certain time steps, representing an increase in attention weights, as shown in the attention plots (y-axis attention weights vs x-axis time steps), were presumed to be video frames capturing important features for decision-making by the DL model. For the time steps at which attention weights peaked, we visualized the gradient weights using Grad-CAM, which revealed differing patterns of motion and behavior between the TD and ASD groups. In the IJA task, which was designed to trigger the participant to initiate social interaction, individuals with TD showed a triadic gaze pattern—gaze shifting from the toy object to the examiner, then back to the toy object—while the individual with ASD failed to gaze on either the toy object or the examiner (eFigure 7A in Supplement 1). In the low-level RJA task, a heatmap around the face and eyes showed that the gaze of the individual with TD on the presented toy object was maintained for long durations, while the gaze of the individual with ASD remained on the toy object only briefly, and then wandered off elsewhere (eFigure 7B in Supplement 1). In the high-level RJA task, individuals with TD immediately turned around to view the poster and then turned back to face the examiner as if seeking approval, while those with ASD showed delayed or no response (eFigure 7C in Supplement 1).

### Interpreting the DL System’s Classification Premises: Hierarchical Clustering

**Figure 2.**
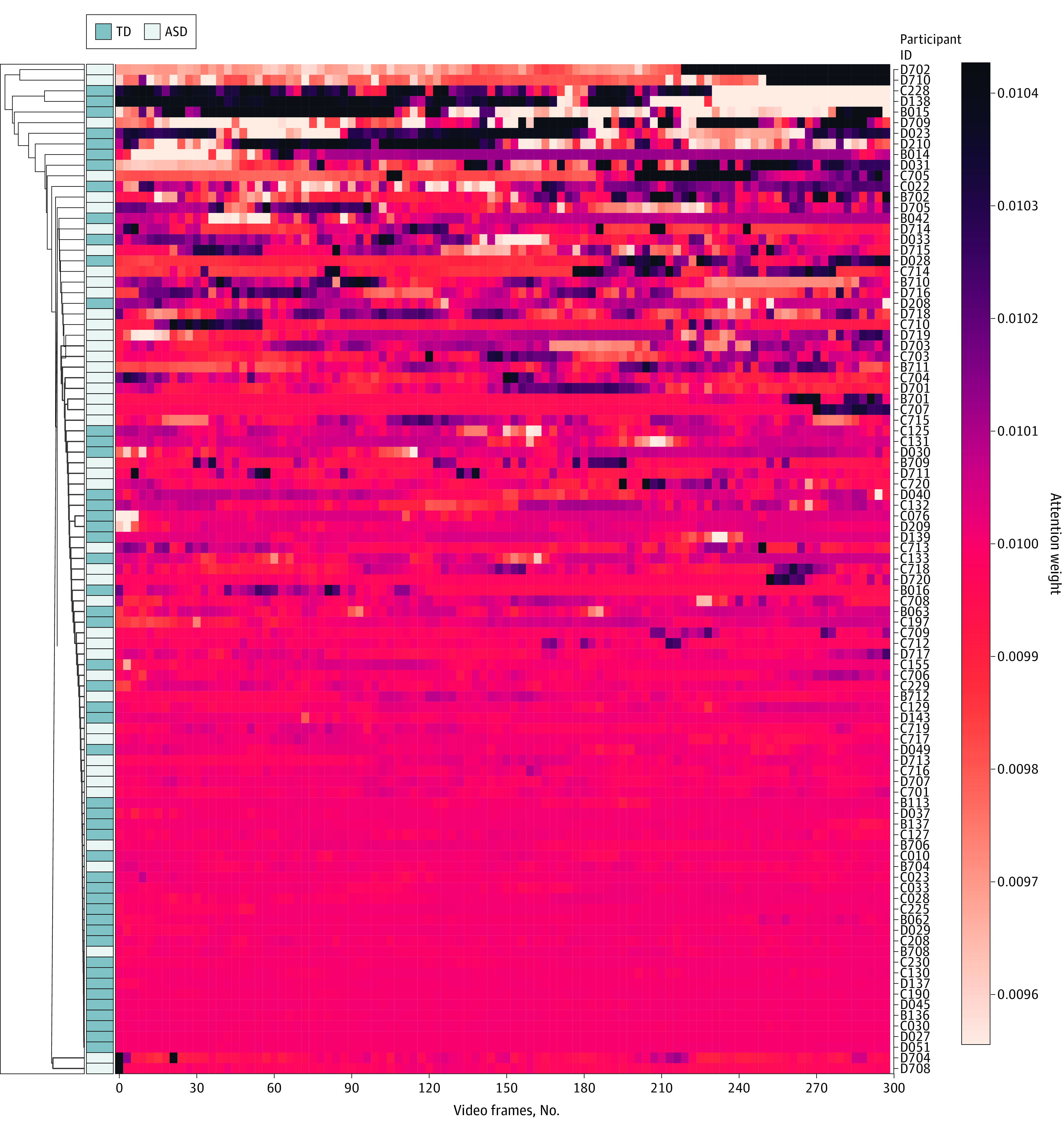
Hierarchically Clustered Heatmaps of Autism Spectrum Disorder (ASD) Detection System The model uses a cluster map of initiation of joint attention (IJA)–based ASD vs typical development (TD). The horizontal axis denotes video frames across time (10 seconds × 30 frames/s = 300 frames). The left vertical axis is the dendrogram, which shows the sequences of merges or splits that occurred during the agglomerative hierarchical clustering. The right vertical axis denotes the different participants included in the testing data set. The cluster map of the IJA-based model shows different patterns of attention weight rise and fall at an individual (patient ID) and diagnostic group (ASD vs TD) level. Darker shades in the heatmap represent an increase in attention weight at a certain time step (video frame), correlated with changes in motion or eye-gaze shifting, as confirmed by the gradient-weighted class activation mapping results. Cluster analysis results, as visualized by the dendrograms, reveal that the IJA task forms clusters, each consisting of either TD or ASD based on heatmap patterns.

**Figure 3.**
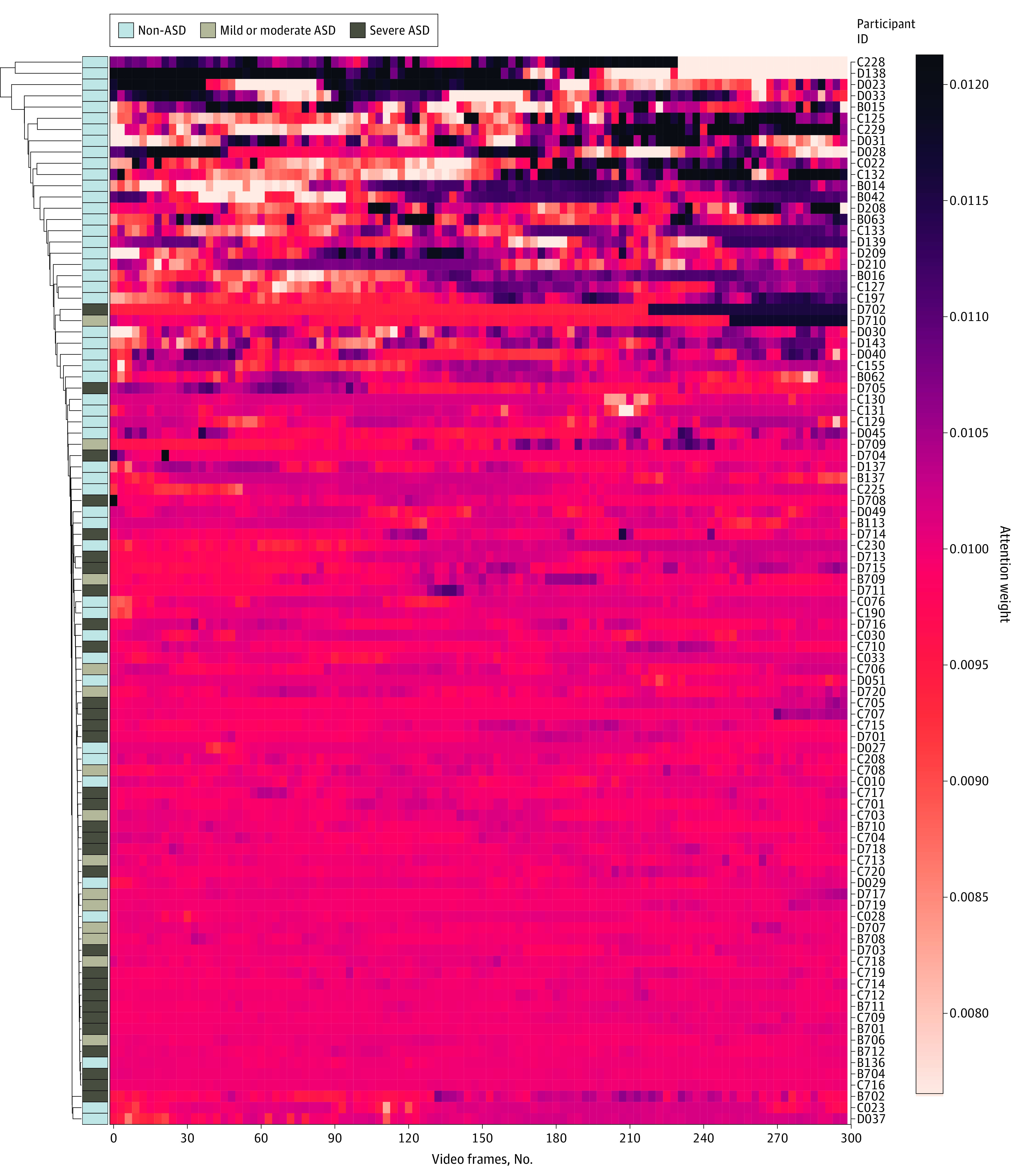
Hierarchically Clustered Heatmaps of Autism Spectrum Disorder (ASD) Symptom Severity Assessment System The model uses a cluster map of initiation of joint attention (IJA)–based non-ASD vs mild or moderate ASD vs severe ASD. The horizontal axis denotes video frames across time (10 seconds × 30 frames/s = 300 frames). The left vertical axis is the dendrogram, which shows the sequences of merges or splits that occurred during the agglomerative hierarchical clustering. The right vertical axis denotes the different participants included in the testing data set. The cluster map of the IJA-based model shows different patterns of attention weight rise and fall at an individual (patient ID) and diagnostic group (non-ASD vs mild or moderate ASD vs severe ASD) level. Darker shades in the heatmap represent an increase in attention weight at a certain time step (video frame), correlated with changes in motion or eye-gaze shifting, as confirmed by the gradient-weighted class activation mapping results. The cluster analysis results, as visualized by the dendrograms, reveal that the IJA task forms clusters, each consisting of either non-ASD, mild or moderate ASD, or severe ASD based on heatmap patterns.

A cluster map hierarchically clusters to order data—in this case, attention weights through the video sequence—by similarity, thereby reorganizing the data for the rows and columns and displaying similar attention weight rise and fall patterns next to one another. Different task types showed different patterns of attention weight peaks between different classes at the data set level. Individuals of the same cluster showed similar peak rise and fall patterns across the time steps, as shown in Figure 2 and Figure 3 and eFigures 9 and 10 in Supplement 1. While all 3 joint attention–based cluster maps sorted ASD vs TD with nearly equal effectiveness (Figure 2 and eFigure 9 in Supplement 1), the IJA-based cluster map (Figure 3) sorted non-ASD vs mild to moderate ASD vs severe ASD more effectively than the RJA-based cluster maps (eFigure 10 in Supplement 1).

## Discussion

To the best of our knowledge, no previous study has demonstrated that DL models for the detection and symptom severity assessment of ASD could be developed using a complex behavioral biomarker such as joint attention. Previous attempts to distinguish ASD from TD using simple gestures^9^ or eye-gaze patterns^35^ were limited by high false-positive rates due to the lack of targeted ASD-related behavioral biomarkers with high discriminatory power or the lack of a replicable method for objectively measuring target behaviors.

In this diagnostic study, our methodical acquisition of input video data for the DL system yielded predictions with high accuracy and precision. By adopting operational definitions of joint attention from well-validated methods,^7,20^ we designed joint attention tasks for easy administration and replicability, eliciting distinctive patterns of social interaction that differed according to diagnosis status and ASD symptom severity. In addition, when acquiring joint attention videos, we monitored the task compliance of every participant at each repeated measure of joint attention. Children with ASD showed qualitatively different behaviors in response to socially salient information. Both ASD and TD showed habituation after 10 repetitions of the IJA task and after 5 repetitions of the RJA tasks. Based on these results, we assume that a joint attention–driven DL model should be trained on videos of the first 5 to 10 repetitions of the same joint attention behavior.

By implementing explainable AI tools, we were able to show that our DL systems made predictions based on salient behavioral differences, such as turning one’s head or shifting one’s eye gaze to view a shared interest better, in the same way trained specialists diagnose ASD, and for assessing the different symptom severities of ASD. Children with TD showed triadic gaze patterns^1,2,7^ during the IJA task, maintained gaze on a visual stimulus during the low-level RJA task, and immediately turned their heads to view a visual stimulus located far away during the high-level RJA task. Children with ASD seldom or never showed triadic gaze patterns during the IJA task, were not able to maintain gaze on visual stimuli during the low-level RJA task, and showed delayed or no response to the examiner’s directing attention to visual stimuli across the room during the high-level RJA task.

Although DL systems trained on any given joint attention task showed high and comparable detection performances in distinguishing ASD vs TD, the IJA-based DL model performed significantly better than the RJA-based models in ASD symptom severity assessment. The RJA tasks, while effective for checking the likelihood of having ASD, may not be sensitive enough to detect subtle motivational nuances pertinent for differentiating one type of ASD from another. Performance of RJA tasks may be associated with development, as model training using the data set of older children showed improved performance. Based on our compliance subanalysis results, it is also plausible that due to early loss of interest during RJA data collection, the DL models could not extract enough feature patterns across individuals with different levels of ASD.

### Limitations

This study has some limitations, including the small sample size and a statistically significant difference in IQ between children with ASD and TD. IQ affects one’s ability to perform behavioral tasks; however, joint attention is a strong trait differentiating ASD from TD even after controlling for IQ.^36^ External validation using a larger sample of IQ-matched cohorts with ASD and TD is still warranted.

## Conclusions

In this diagnostic study, we developed DL models for identifying ASD and differentiating levels of ASD symptom severity. We believe our research opens possibilities for gathering large data sets on behavioral biomarkers through standardized video data acquisition setup amenable to computer vision and DL and applicable to a wide range of neuropsychiatric conditions.^37,38^ Moreover, our findings suggest that our method may be a good alternative for assisting clinicians in making well-informed referrals to a child development specialist or in making ASD diagnosis.

## Notes

Supplement 1. **eFigure 1.** Study Overview Diagram **eFigure 2.** Joint Attention Types and Operational Definitions **eFigure 3.** Joint Attention Task—Video Data Acquisition Setup **eFigure 4.** Deep Learning System Architecture **eFigure 5.** Compliance Score Analyses **eFigure 6.** Performance of Deep Learning System by Age Group (<48 vs ≥48 mo) **eFigure 7.** Gradient-Weighted Class Activation Maps of Joint Attention Videos **eFigure 8.** Examples of Attention Plots **eFigure 9.** Hierarchically Clustered Heatmaps of ASD Detection System **eFigure 10.** Hierarchically Clustered Heatmaps of ASD Symptom Severity Assessment System **eTable 1.** Compliance Score Metric **eTable 2.** Performance of Deep Learning System for 3 Joint Attention Types—Training and Validation
eReferences

Supplement 2.
Data Sharing Statement
