# Error in Corresponding Author Contact Information

**DOI:** 10.1001/jamanetworkopen.2023.24944

**Published:** 2023-07-07

In the Original Investigation titled “Development and Validation of a Joint Attention–Based Deep Learning System for Detection and Symptom Severity Assessment of Autism Spectrum Disorder,”^1^ published May 25, 2023, certain components of Soon-Beom Hong’s contact information were incorrect. This article has been corrected.
