# Supplementary material for: Development and Validation of a Joint Attention–Based Deep Learning System for Detection and Symptom Severity Assessment of Autism Spectrum Disorder
Source: JAMA Netw Open. 2023 May 25;6(5):e2315174. doi: 10.1001/jamanetworkopen.2023.15174 (PMC10214037; doi:10.1001/jamanetworkopen.2023.15174)
Supplement: Supplement 1. — eFigure 1. Study Overview Diagram eFigure 2. Joint Attention Types and Operational Definitions eFigure 3. Joint Attention Task—Video Data Acquisition Setup eFigure 4. Deep Learning System Architecture eFigure 5. Compliance Score Analyses eFigure 6. Performance of Deep Learning System by Age Group (<48 vs ≥48 mo) eFigure 7. Gradient-Weighted Class Activation Maps of Joint Attention Videos eFigure 8. Examples of Attention Plots eFigure 9. Hierarchically Clustered Heatmaps of ASD Detection System eFigure 10. Hierarchically Clustered Heatmaps of ASD Symptom Severity Assessment System eTable 1. Compliance Score Metric eTable 2. Performance of Deep Learning System for 3 Joint Attention Types—Training and Validation eReferences [file jamanetwopen-e2315174-s001.pdf]

Supplementary Online Content

Ko C, Lim JH, Hong J, Hong SB, Park YR. Development and validation of a joint attention–based deep learning system for detection and symptom severity assessment of autism spectrum disorder. *JAMA Netw Open*. 2023;6(5):e2315174. doi:10.1001/jamanetworkopen.2023.15174

eFigure 1. Study Overview Diagram
eFigure 2. Joint Attention Types and Operational Definitions
eFigure 3. Joint Attention Task—Video Data Acquisition Setup
eFigure 4. Deep Learning System Architecture
eFigure 5. Compliance Score Analyses
eFigure 6. Performance of Deep Learning System by Age Group (<48 vs ≥48 mo)
eFigure 7. Gradient-Weighted Class Activation Maps of Joint Attention Videos
eFigure 8. Examples of Attention Plots
eFigure 9. Hierarchically Clustered Heatmaps of ASD Detection System
eFigure 10. Hierarchically Clustered Heatmaps of ASD Symptom Severity Assessment System
eTable 1. Compliance Score Metric
eTable 2. Performance of Deep Learning System for 3 Joint Attention Types—Training and Validation
eReferences

This supplemental material has been provided by the authors to give readers additional information about their work.

© 2023 Ko C et al. *JAMA Network Open*.

eFigure 1. Study Overview Diagram

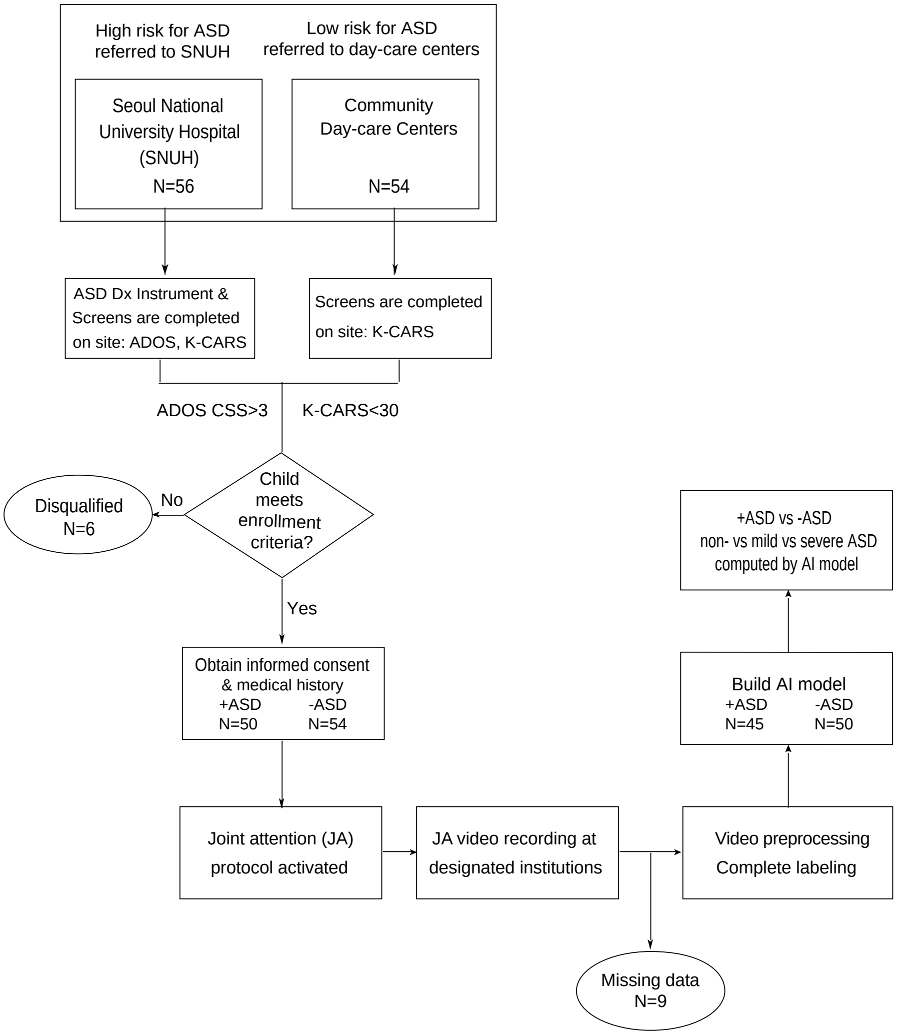

Children with 1) sensory or motor impairment that preclude sitting or viewing the stimuli presented by an examiner, 2) severe behavioral problems, such as self-harming and/or aggression, and 3) comorbid psychiatric conditions, such as attention-deficit/hyperactivity disorder, were excluded from the study. Those who were at high risk for ASD and those with low risk (no previous developmental delay or psychiatric history) were recruited from different institutions.

ASD participants (+ASD) were finally selected if they were diagnosed as ASD by a child psychiatrist according to the Diagnostic and Statistical Manual of Mental Disorders-V[1] criteria with confirmatory Autism Diagnostic Observation Schedule-2 (ADOS-2) score.[2,3] Those with an ADOS-2 CSS[4] <3 (“non-ASD”) were excluded. Five ASD participants failed to complete all study assessments and were therefore excluded from analysis. As a result, 45 of the 56 (80.3%) high risk children were selected for ASD group (+ASD). Typically developing participants (-ASD) were considered for inclusion if they showed negative screening results based on Korean Childhood Autism Rating Scale-2nd Edition (K-CARS-2).[5,6] While all individuals initially enrolled into typically developing (-ASD) group showed K-CARS<30 (100%), four individuals failed to complete all study measures; hence, 50 of the 54 (92.6%) were finally included for analysis.

Abbreviations: ADOS, Autism Diagnostic Observation Scale; ADOS CSS, ADOS Total Severity Calibrated Scores; AI, Artificial Intelligence; ASD, Autism Spectrum Disorder; JA, Joint Attention; K-CARS, Korean Childhood Autism Rating Scale

© 2023 Ko C et al. *JAMA Network Open*.

eFigure 2. Joint Attention Types and Operational Definitions

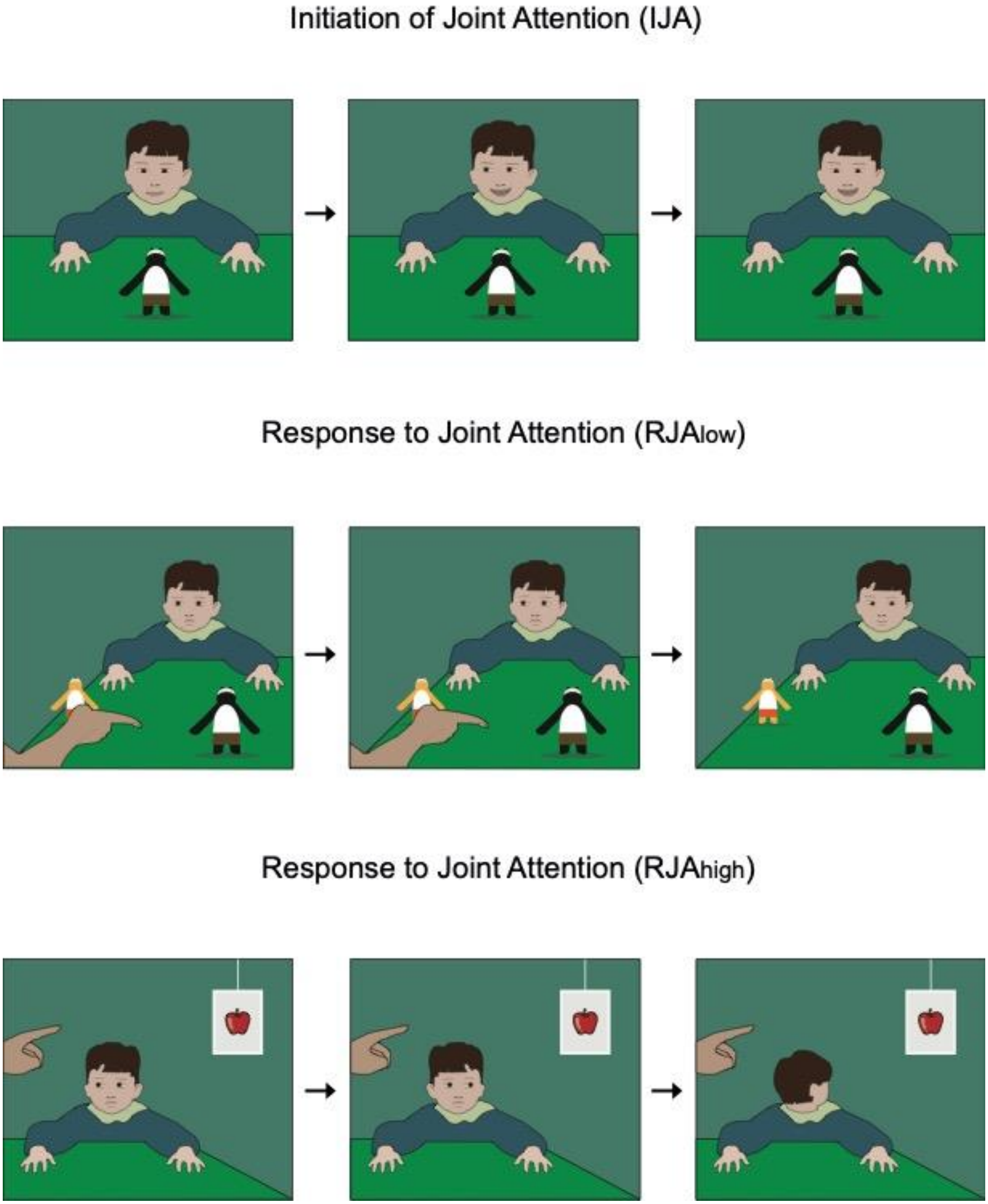

A visual representation is provided for the operational definitions of the three different types of joint attention tasks.

© 2023 Ko C et al. *JAMA Network Open*.

Joint attention task procedures:

Initiation of Joint Attention. A rotation of age- and culture-appropriate test toys, which were selected following the guidelines of the Autism Diagnostic Observation Schedule-2[2,3] and Mundy's Early Social Communication Scales manual[7] (10 different types or shapes of similar size: width × length × height = 3 cm × 5 cm × 3 cm), was placed along the midline, 70 cm away from the edge of the table at which the child was seated. If the toy was placed too close to the child, they would simply pick it up and play with it without making any effort to interact with the examiner. A trained examiner was seated adjacent to where the toy was placed, such that the child could see the examiner's face by making an approximately 45-degree head turn or by shifting their gaze considerably to their right. The experimental process was as follows: the examiner placed a toy at the designated spot, waited for 30 seconds, and simply faced the child without providing verbal instructions. Once the child initiated joint attention by shifting their gaze from the toy to the examiner and back to the toy (sometimes also pointing at the toy), the examiner was asked to shift their gaze or turn their head to the toy to match the child's response. This task was repeated once more with the same toy after a 30 second pause, and then a different toy was introduced. The order of the toy presentation was pseudo-randomized across participants. If the children failed to show any interest in the toy after 30 seconds, the examiner could use an alternate toy.

Response to Joint Attention. Response to joint attention (RJA) tasks were designed to observe whether a child would direct and maintain their attention on an object to which the examiner pointed with their index finger. Depending on the distance between the examiner's index finger and the object, the RJA tasks were further divided into RJA_{low} (near) and RJA_{high} (far) tasks, where RJA_{low} involved the examiner pointing to toy objects on the table and RJA_{high} involved the examiner pointing to poster pictures on the walls. The RJA_{low} task utilized stimuli similar to that of the initiation of joint attention task, except that two toy objects (one as a stimulus and the other as a distractor) were used. Four posters depicting a child-friendly image (a car, butterfly, bananas, and puppy) covering half of an A4 sheet were pasted onto three walls—left, right, and behind—with respect to where the child participant was seated. Each poster was approximately 100 cm from the child's position.

© 2023 Ko C et al. *JAMA Network Open*.

eFigure 3. Joint Attention Task—Video Data Acquisition Setup

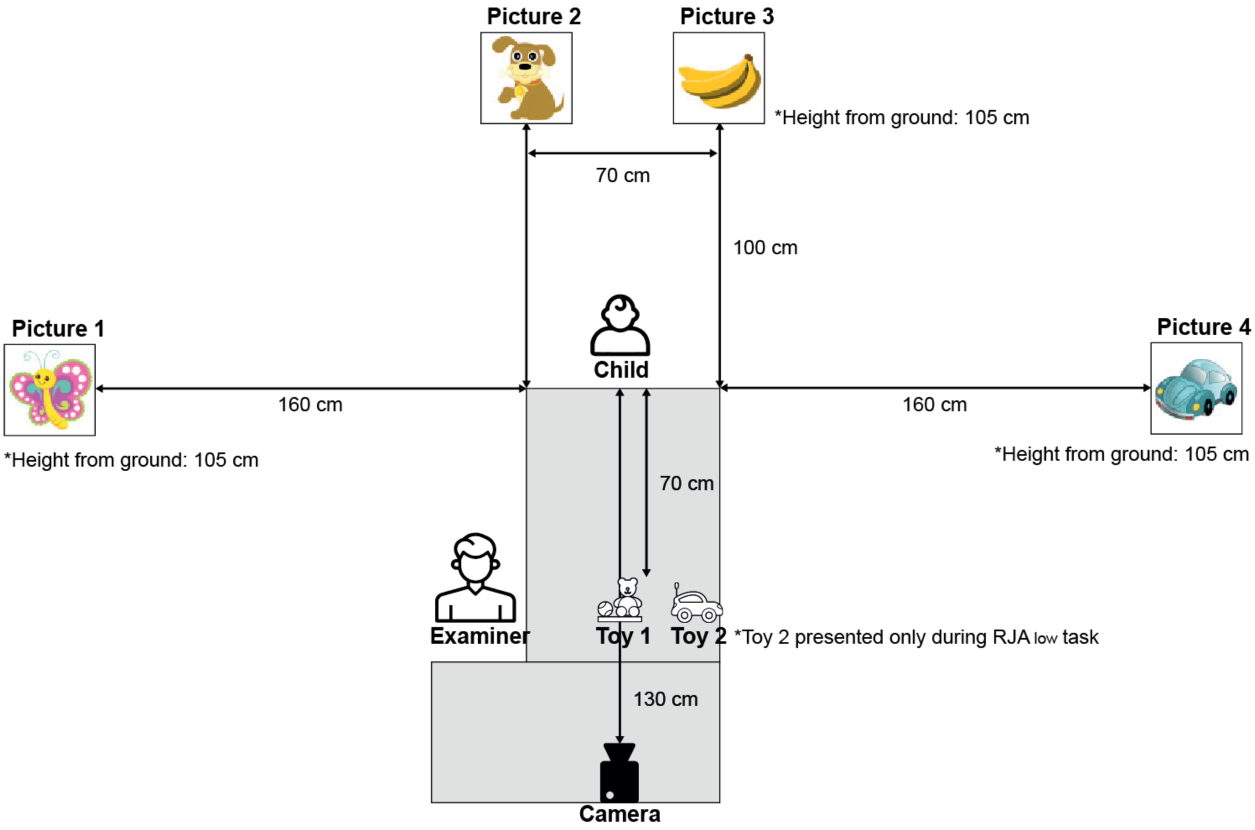

Setup for joint attention experiments based on our protocol. Initiation of joint attention (IJA) tasks required use of only toy 1, while response to joint attention tasks, low (RJA_{low}), required the use of toy 1 and toy 2 (distraction), and response to joint attention tasks, high (RJA_{high}), required use of pictures 1-4.

© 2023 Ko C et al. *JAMA Network Open*.

eFigure 4. Deep Learning System Architecture

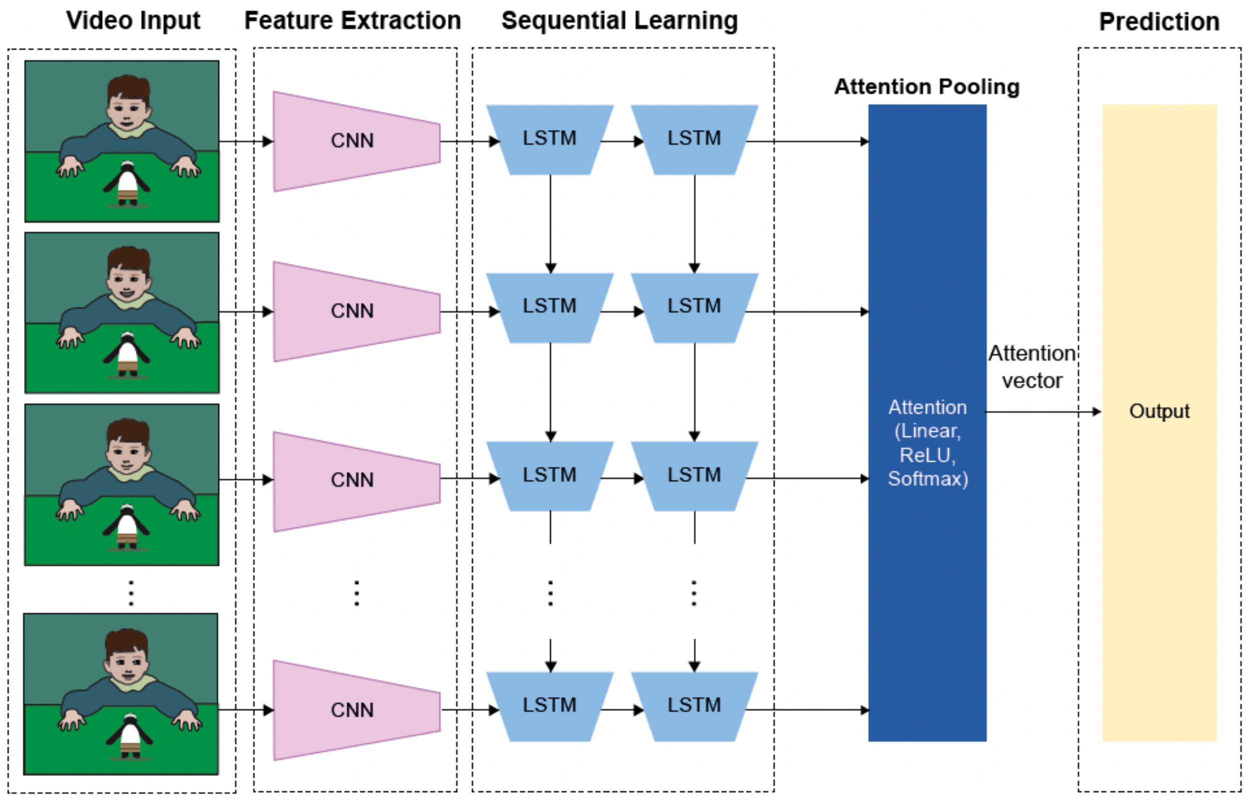

Convolutional neural network (CNN)–Long Short-Term Memory (LSTM)–Attention-based deep learning system used for classifying autism spectrum disorder (ASD) vs typical development (TD) and non-ASD vs mild–moderate ASD vs severe ASD using video input.

In the following section, we describe the stepwise development of the deep-learning prediction system:

Step 1: Video data for each trial per participant are gathered, pre-processed to remove background, center-cropped, and resized to 224 × 224 pixels. For IJA task, input size of one video is 224x224x300 (30 frames/second x 10 seconds). For RJA task, input size of one video is 224x224x150 (30 frames/second x 5 seconds). The video dataset is split 8:1:1 for training, validation, and testing of deep learning model. To minimize sampling bias and overfitting of the model, we used 10-fold cross validation technique. True label for ASD vs TD model was the diagnosis status (TD:0, ASD: 1) confirmed by a child psychiatrist of 10+ years of experience. Korean Childhood Autism Rating Scale-2nd Edition (K-CARS 2) cut-off scores for non-ASD (0), mild-moderate ASD (1) vs severe ASD (2)[5,6] were used as true-label values for ASD symptom severity.

Step 2: A CNN was employed to extract salient behavioral features. For efficient training, we fine-tuned an ImageNet-pre-trained ResNet-18[8], freezing the last residual block[9] so that the last convolution layers could be trained on our own video dataset.

Step 3: Two unilateral layers of LSTM were incorporated to improve processing and predictions using sequential (time-dependent) video data.

Step 4: The attention layer was incorporated so that our deep learning system would be able to localize salient information when making predictions. The logic of having an 'attention mechanism' in our deep learning system as opposed to a simple LSTM-based model was that it would be able to pay attention to particular areas or objects rather than treating the whole image equally.

Step 5: The last layer of the deep learning system outputs the probabilities *p*(ASD) and *p*(TD) for the two-class classification and the probabilities *p*(non-ASD), *p*(mild–moderate ASD), and *p*(severe ASD) for the three-class classification.

Codes are available at https://github.com/DigitalHealthcareLab/JointAttention.git

© 2023 Ko C et al. *JAMA Network Open*.

eFigure 5. Compliance Score Analyses

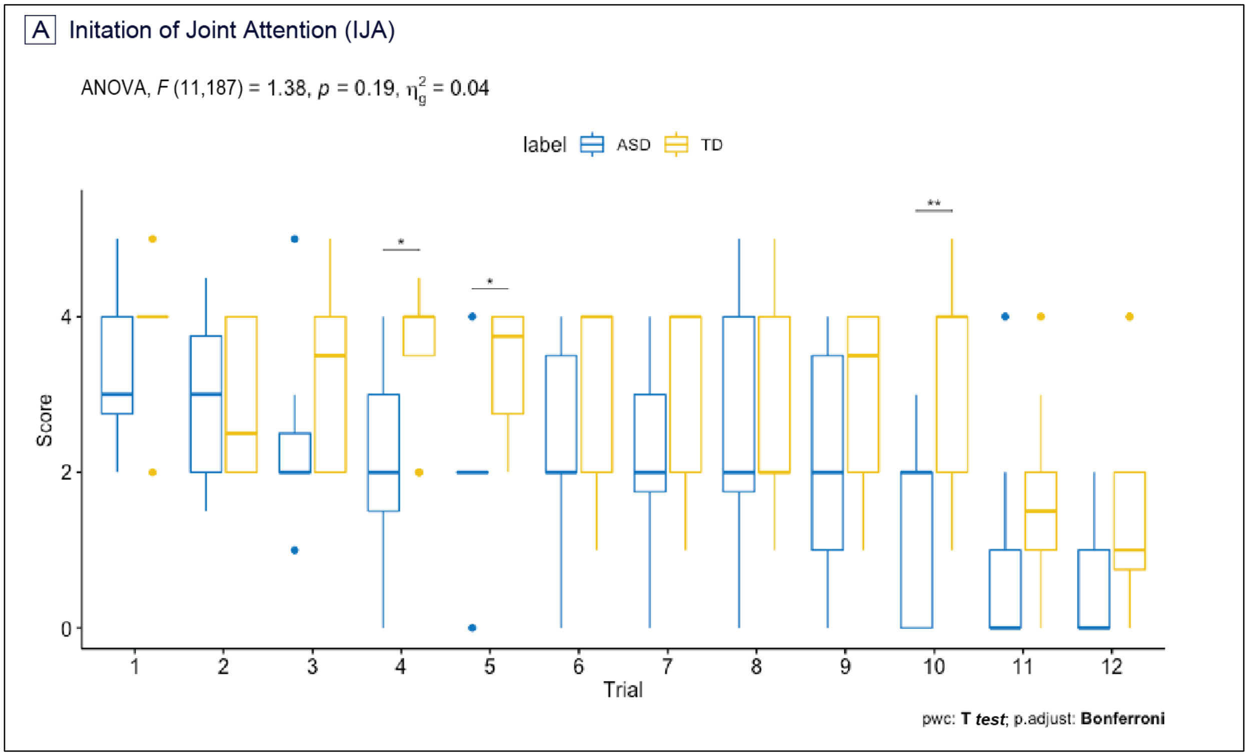

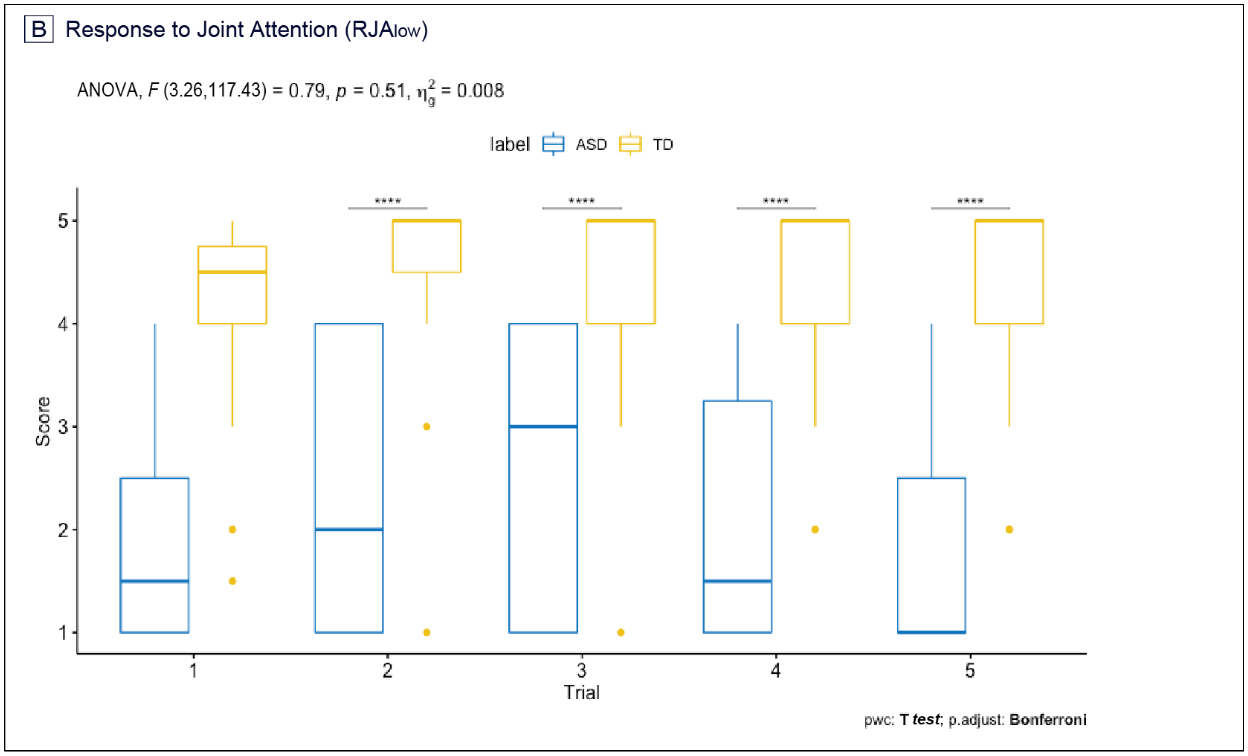

© 2023 Ko C et al. *JAMA Network Open*.

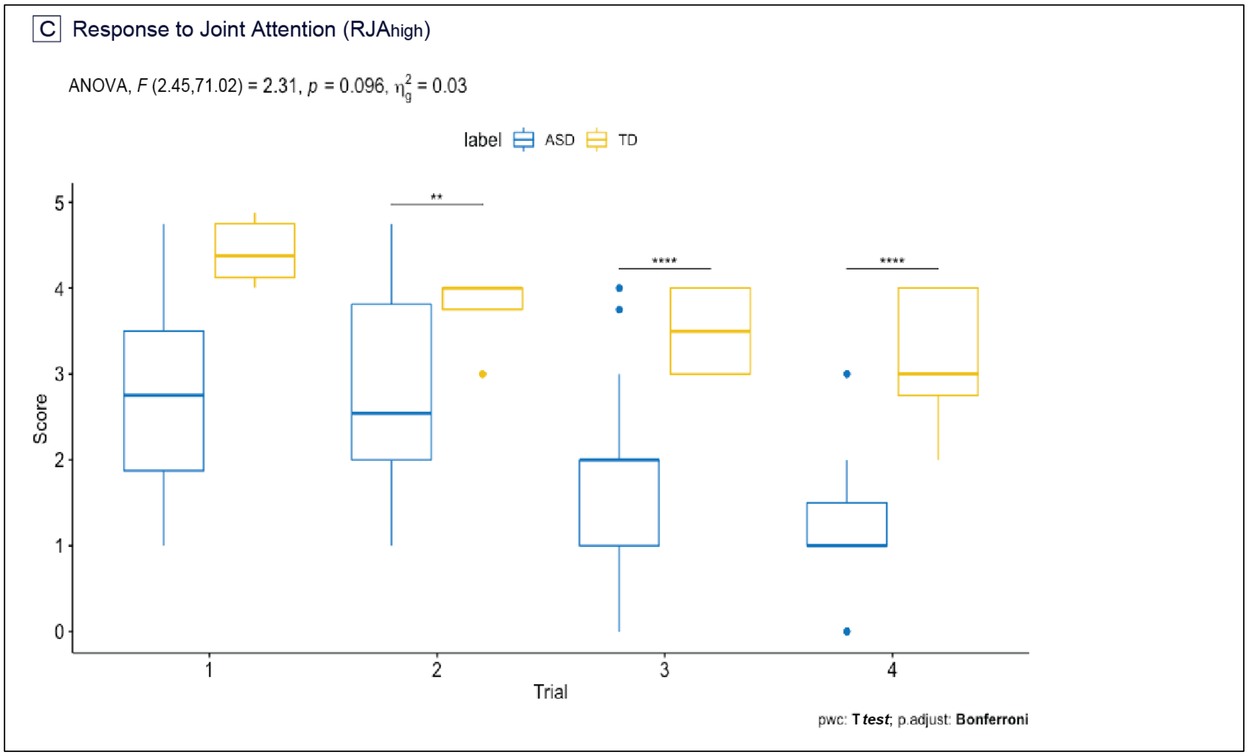

Joint attention trials and number of successfully elicited joint attention behaviors for each task type: initiation of joint attention (IJA), response to joint attention, low (RJA_{low}), and response to joint attention, high (RJA_{high}).

Task compliance (or attentiveness) according to diagnostic group (ASD vs TD) for the three joint attention tasks is depicted by trial vs. score box plots. To explore the effects of both diagnostic group and time ('trial') on compliance ('score'), we performed a two-way mixed analysis of variance (ANOVA), which showed that there were no statistically significant two-way interaction effects of the diagnostic group (ASD vs TD) and the number of repeated trials on the compliance score (0–5) during IJA tasks (F (11, 187) = 1.38, p = 0.186). There was a statistically significant effect of the diagnostic group on the compliance score during IJA tasks in trials 4 (p = 0.0206), 5 (p = 0.0128), and 10 (p = 0.00371). A paired t-test for the trial variable showed that compliance in trials 11 and later was significantly lower than that in the previous 10 trials (p < 0.05). There were no statistically significant effects of a two-way interaction between the diagnostic group and the number of trials on the compliance score during either RJA_{low} task (F (3.26, 117.43) = 0.79, p = 0.51) or RJA_{high} task (F (2.45, 71.02) = 2.31, p = 0.096). However, there was a statistically significant effect of the diagnostic group on the compliance score for RJA_{low} and RJA_{high} tasks across all trials (p < 0.05). Significant differences by group effect (ASD vs TD) are indicated with asterisks.

© 2023 Ko C et al. *JAMA Network Open*.

eFigure 6. Performance of Deep Learning System by Age Group (<48 vs ≥48 mo)

(A)

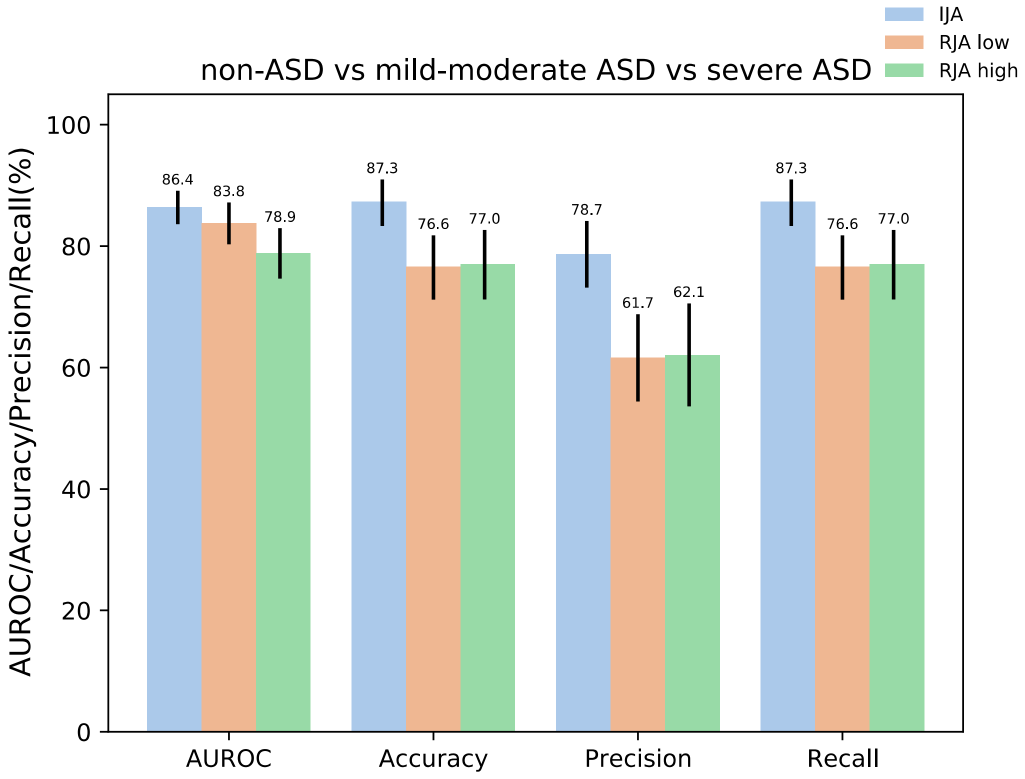

© 2023 Ko C et al. *JAMA Network Open*.

(B)

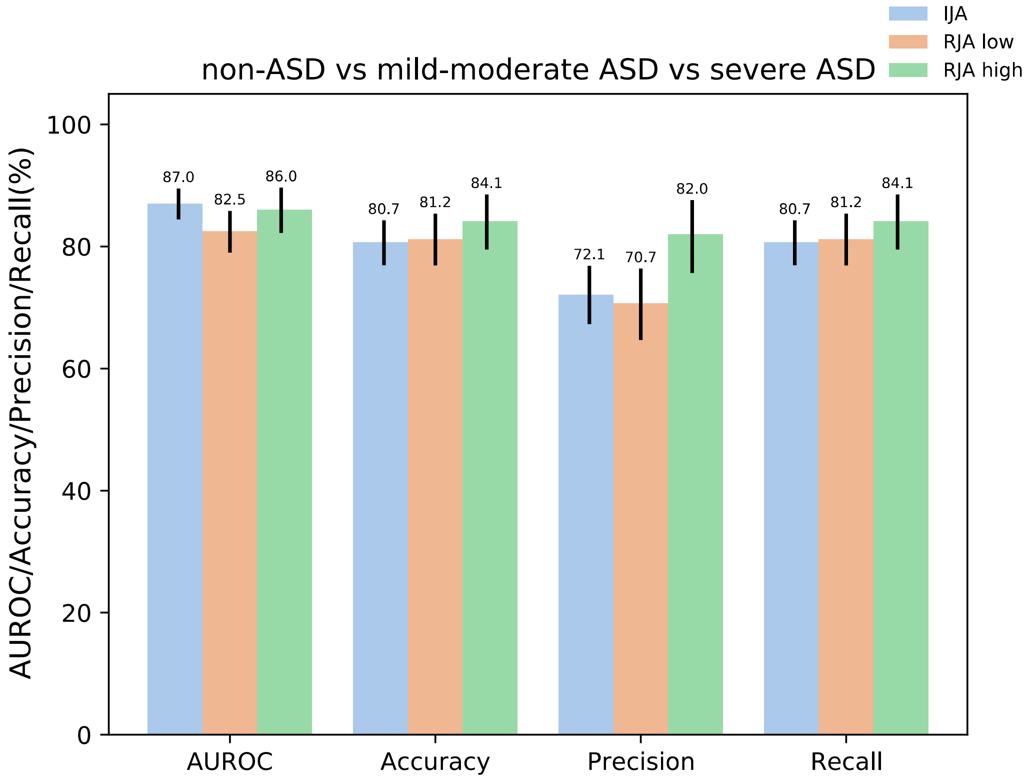

(A) Bar graphs of ASD Symptom Severity Assessment Model's (24-47 months) prediction AUROC, accuracy, precision, and recall, using IJA (Blue), RJA_{low} (Orange), and RJA_{high} (Green) testing datasets. (B) Bar graphs of ASD Symptom Severity Assessment Model's (48-72 months) prediction AUROC, accuracy, precision, and recall, using IJA (Blue), RJA_{low} (Orange), and RJA_{high} (Green) testing datasets.

* The mean and 95% confidence intervals (error bars) were calculated from all ASD detection (A) and symptom severity assessment (B) models based on 10-fold cross-validation.

Abbreviations: ASD, autism spectrum disorder; AUROC, area under the receiver operating characteristic curve; IJA, initiation of joint attention; RJA_{low}, response to low joint attention level; RJA_{high}, response to high joint attention level

© 2023 Ko C et al. *JAMA Network Open*.

eFigure 7. Gradient-Weighted Class Activation Maps (GradCAM) of Joint Attention Videos

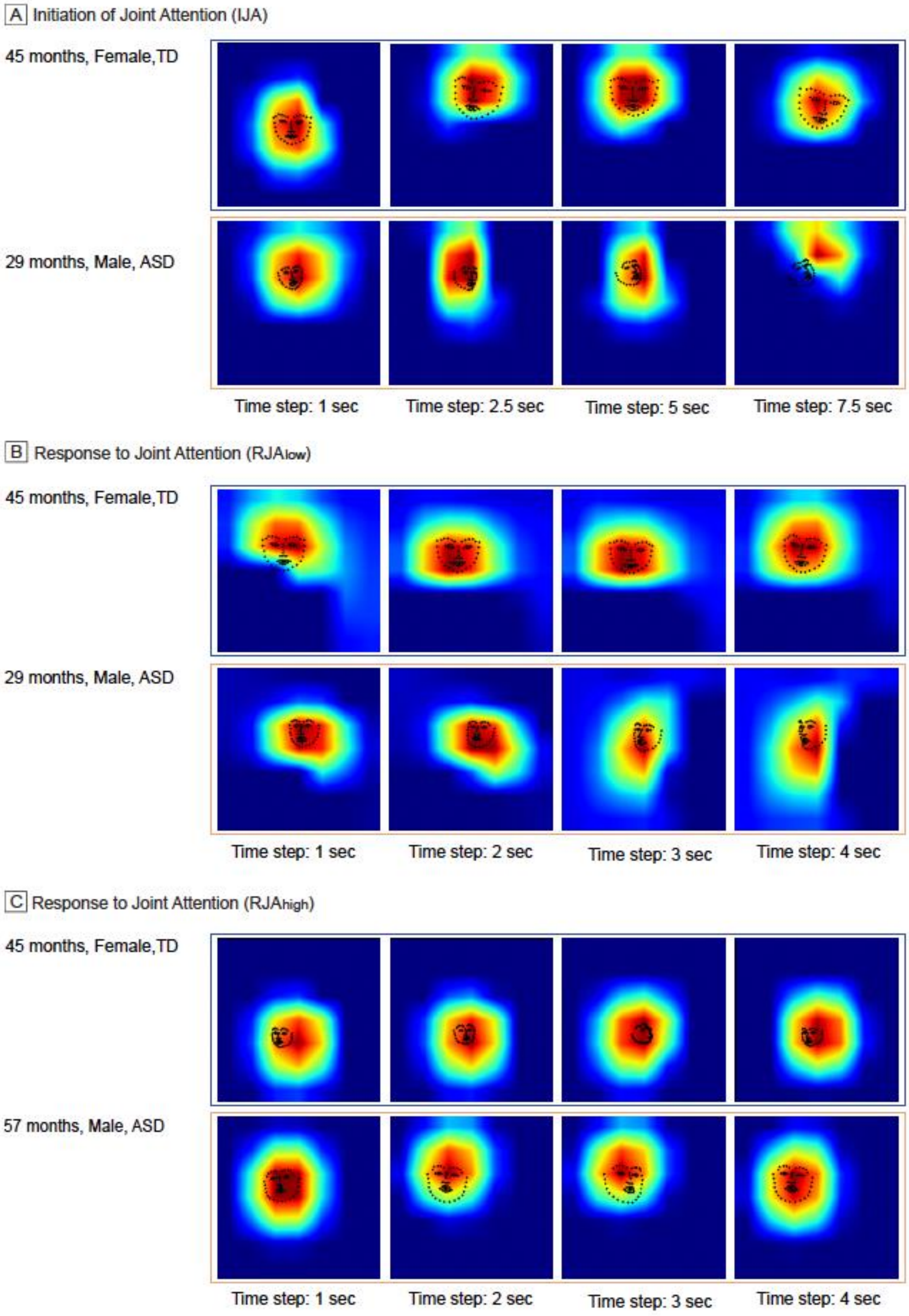

© 2023 Ko C et al. *JAMA Network Open*.

A. GradCAM applied to IJA video of one ASD and one TD participant, B. GradCAM applied to RJA_{low} video of one ASD and one TD participant, C. GradCAM applied to RJA_{high} video of one ASD and one TD participant.

Illustration of GradCAM applied to joint attention video frames across time. Overlaid visual explanation heatmaps highlight key areas such as the participant's face and areas of interest to which the participant shifts their gaze. The video collection was conducted at 30 frames/second intervals. The length of an IJA video is 10 seconds and that of a RJA video is 5 seconds. Although 2D RGB videos were used for training and validation of the deep learning system, to preserve the privacy of the participants, we present the GradCAM results with facial landmarks—using a facial recognition library called dlib.[10] The GradCAM results suggest that the deep learning system predicted ASD vs TD based on head-turn or gaze-shift patterns, which are different by individual as well as by joint attention task type.

© 2023 Ko C et al. *JAMA Network Open*.

eFigure 8. Examples of Attention Plots

(A)

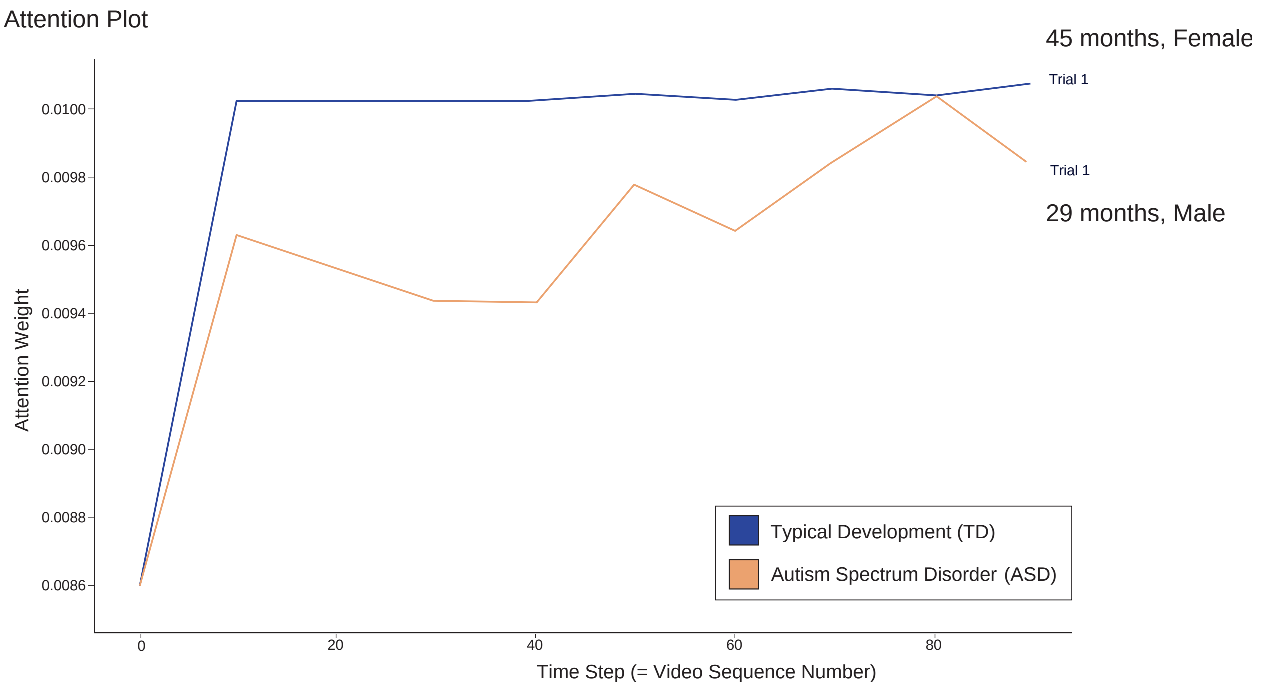

(B)

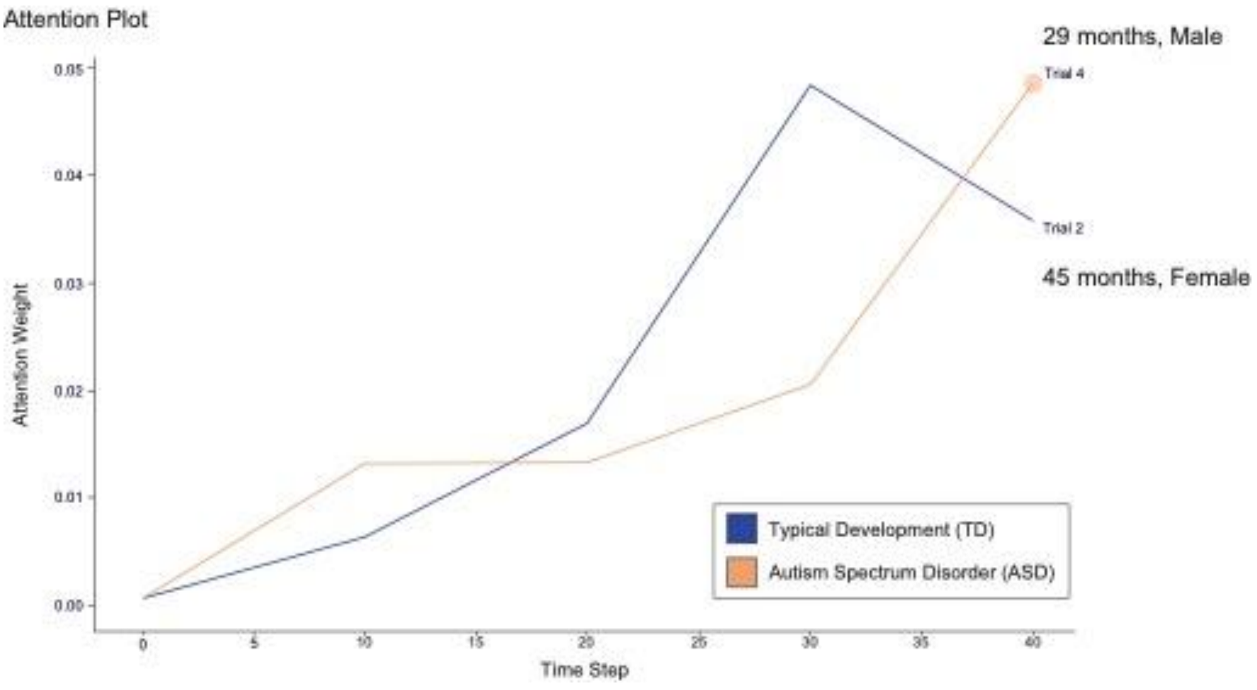

© 2023 Ko C et al. *JAMA Network Open*.

(C)

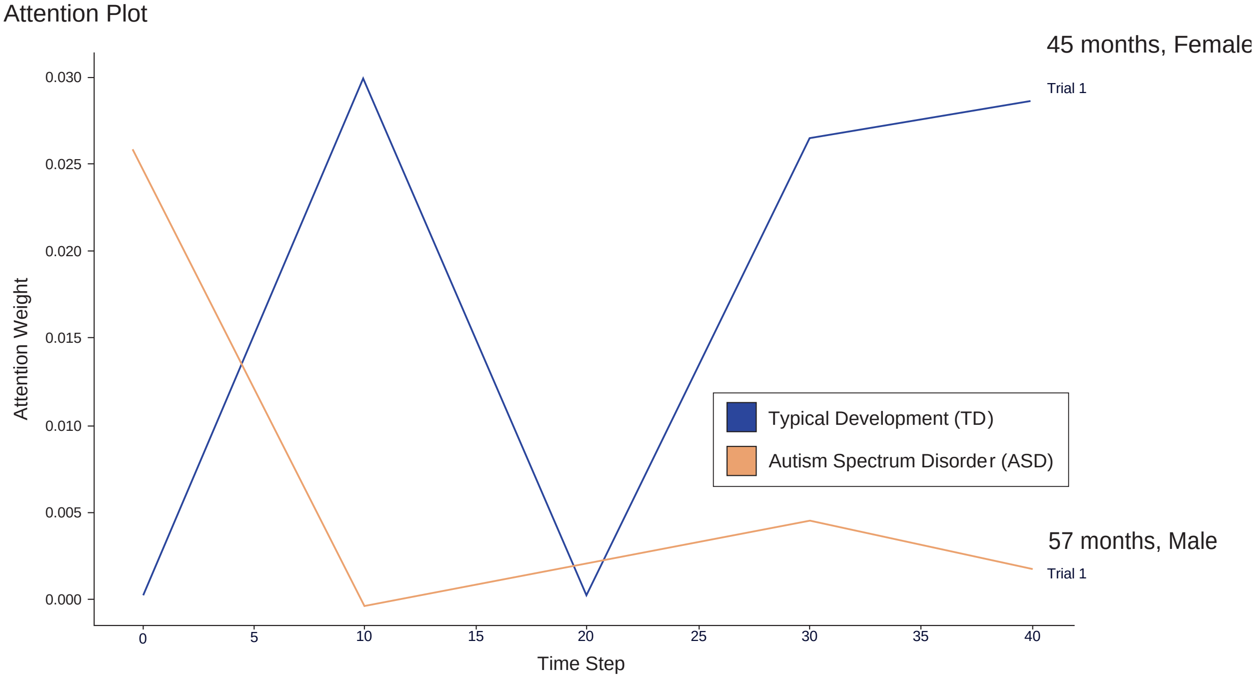

The two lines, representing attention plots of a TD (blue line) or an ASD (yellow line) participant, were plotted using the attention weights acquired from (A) IJA-, (B) RJA_{low}-, (C) RJA_{high}-based ASD vs TD models. During training of each model, attention weights were calculated and accumulated in the "attention layer".

An attention plot visualizes changes in attention weights (y-axis) across time steps (x-axis) of a single video capturing a child engaging in a single trial of joint attention task. The purpose of drawing an attention plot is to get a sense of which video frames ("images") at which time point along the whole length of the video the deep learning model considered more important than others in making the prediction (ASD vs TD). The three attention plots above are attention plots for IJA, RJA_{low}, and RJA_{high} task, in that specific order. Per joint attention task, we drew attention plots for one TD and one ASD for comparison. Video data collection was conducted at 30 frames/second intervals, and video sequence lengths for initiation of the joint attention task and response to joint attention tasks were 300 frames (= 10 seconds) and 150 frames (= 5 seconds), respectively. For better visualization, we selected every three frames, rendering 100 frames (time steps) and 50 frames (time steps).

Abbreviations: ASD, autism spectrum disorder; IJA, initiation of joint attention; RJA_{low}, response to low joint attention level; RJA_{high}, response to high joint attention level; TD, typical development

© 2023 Ko C et al. *JAMA Network Open*.

eFigure 9. Hierarchically Clustered Heatmaps of ASD Detection System

(A)

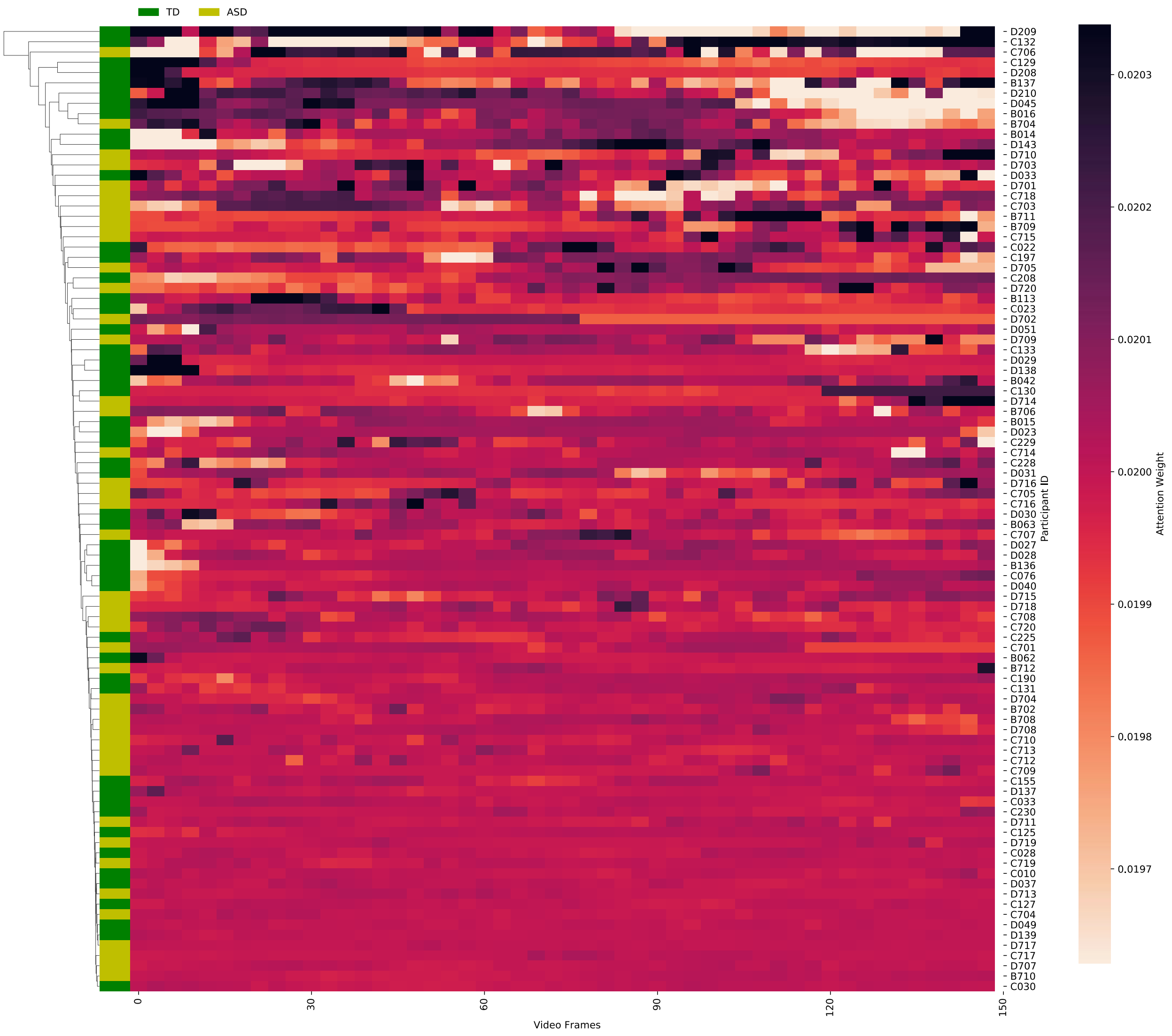

© 2023 Ko C et al. *JAMA Network Open*.

(B)

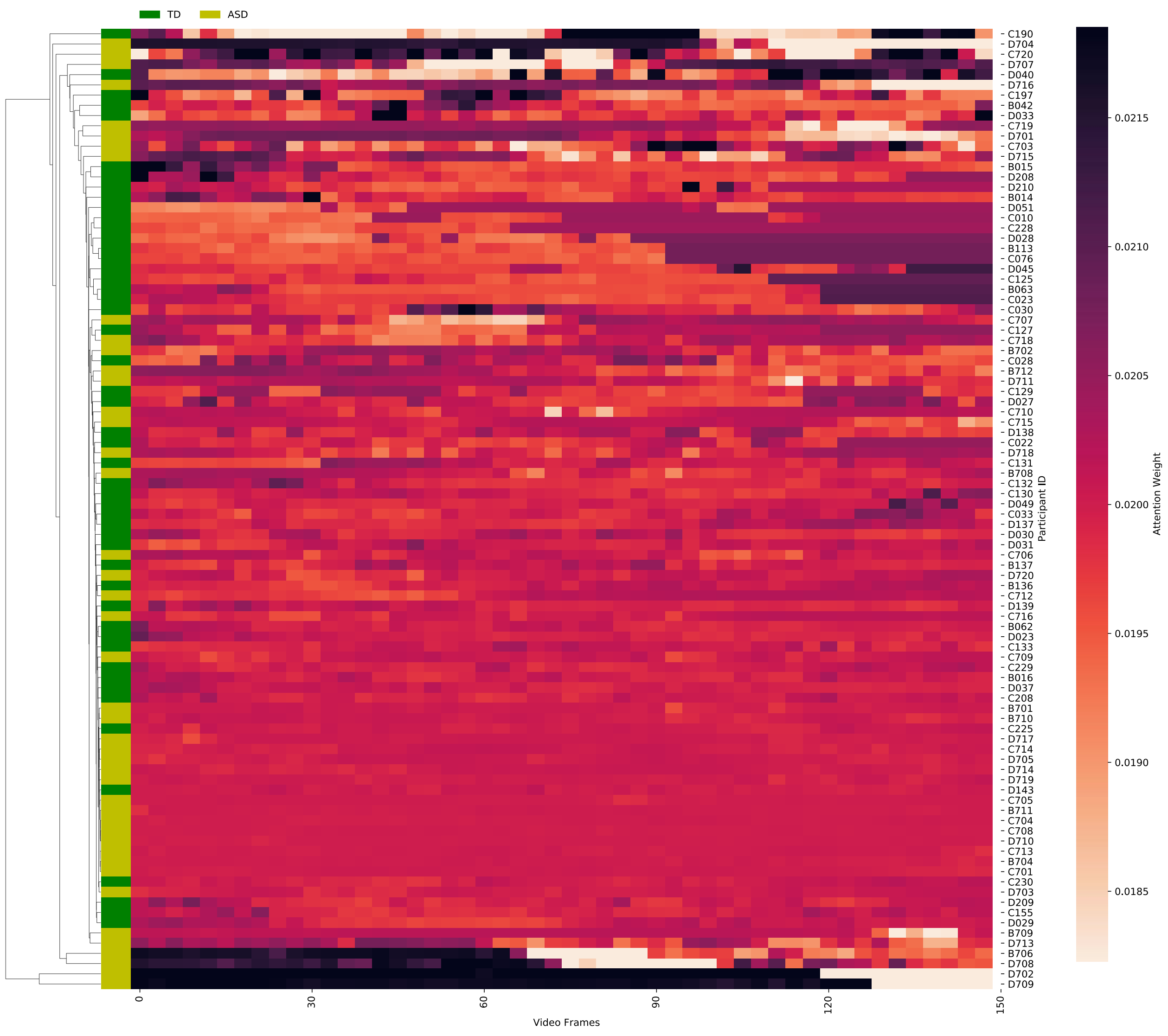

© 2023 Ko C et al. *JAMA Network Open*.

A. Cluster map of RJA_{low}-based ASD vs TD model, B. Cluster map of RJA_{high}-based ASD vs TD model.

A cluster map uses hierarchical clustering to order attention weights (each weight represented by a heatmap) through the video frames by similarity, thereby reorganizing the data for the rows and columns and displaying similar rise (darker shade) and fall (lighter shade) in attention weight patterns next to one another. The horizontal axis denotes video frames across time (5 seconds x 30 frames/second = 150 frames). Left vertical axis is the dendrogram, which shows the sequences of merges or splits that occurred during the agglomerative hierarchical clustering. Right vertical axis denotes the different participants included in the testing dataset. Cluster maps of RJA_{low} and RJA_{high}-based models both show different patterns of attention weight rise/fall at an individual (patient ID) and diagnostic group (ASD vs TD) level. Darker shades in the heatmap, representing an increase in attention weight at a certain time step (video frame), correlated with changes in motion or eye-gaze shifting, as confirmed by the Grad-CAM results. Cluster analysis results, as visualized by the dendrograms, reveal that response to joint attention tasks form clusters each consisted of either TD (green) or ASD (yellow) based on heatmap patterns.

Abbreviations: ASD, autism spectrum disorder; IJA, initiation of joint attention; RJA_{low}, response to low joint attention level; RJA_{high}, response to high joint attention level; TD, typical development

© 2023 Ko C et al. *JAMA Network Open*.

eFigure 10. Hierarchically Clustered Heatmaps of ASD Symptom Severity Assessment System

(A)

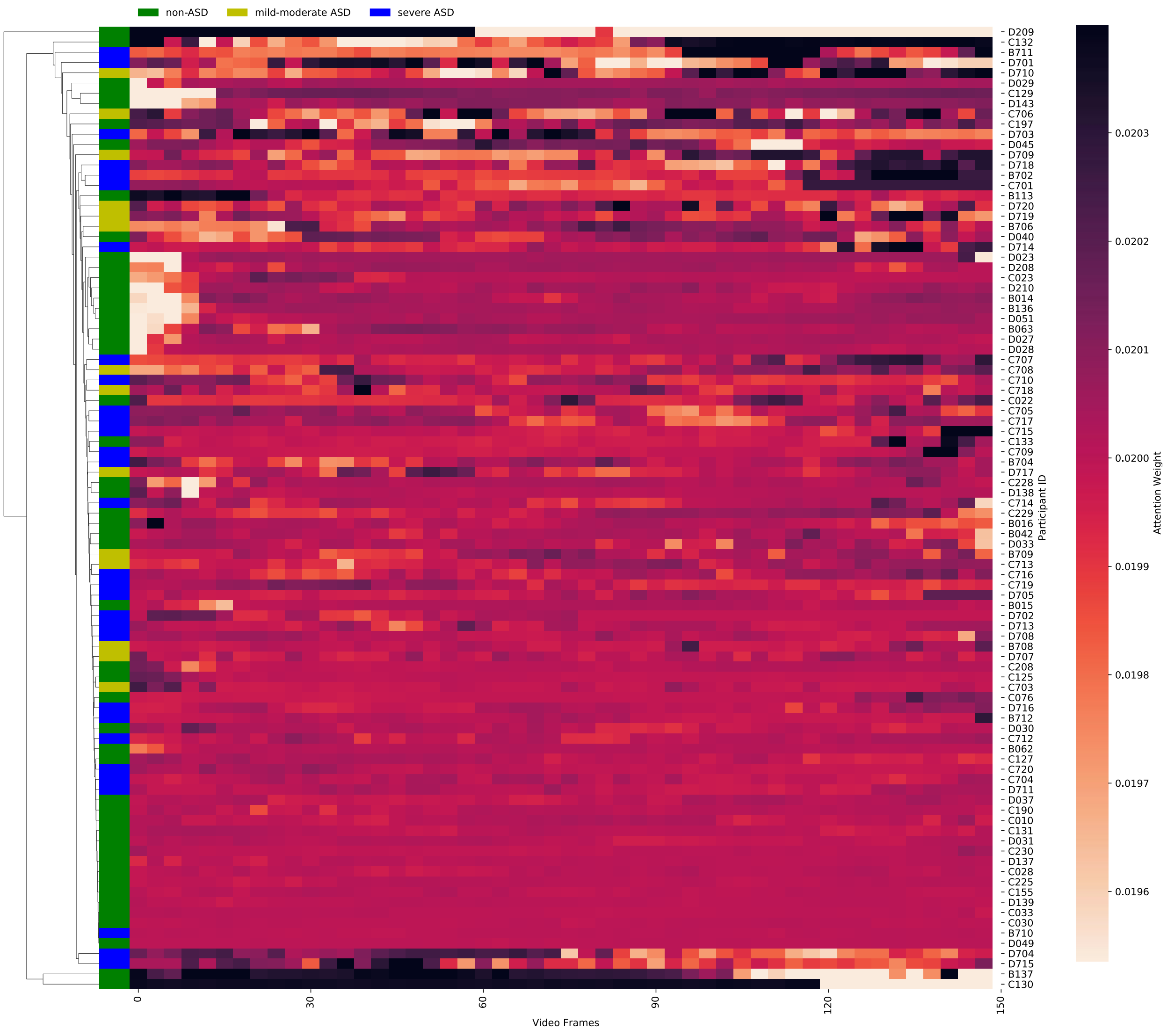

© 2023 Ko C et al. *JAMA Network Open*.

(B)

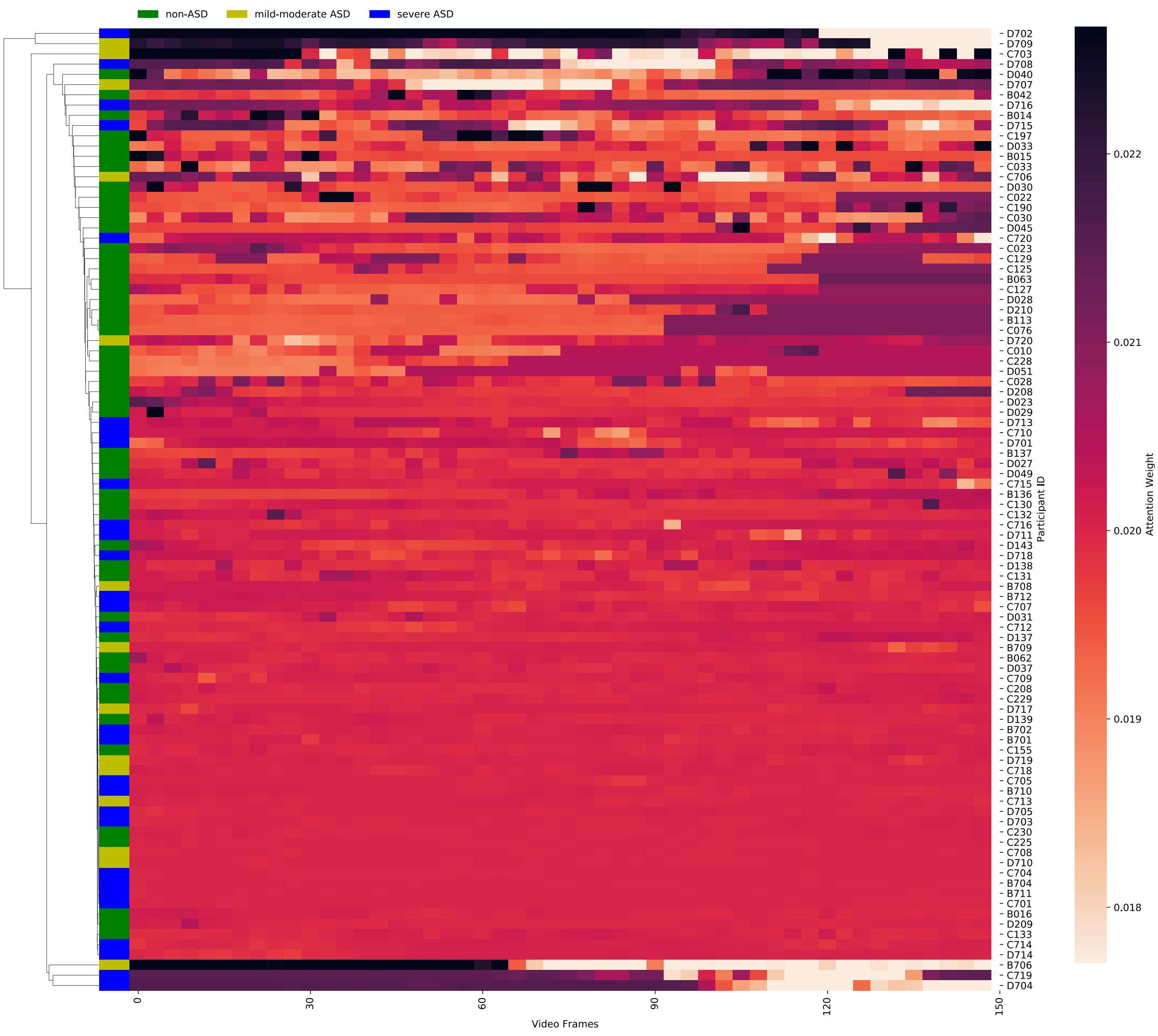

© 2023 Ko C et al. *JAMA Network Open*.

A. Cluster map of RJA_{low}-based non-ASD vs mild-moderate ASD vs severe ASD model, B. Cluster map of RJA_{high}-based non-ASD vs mild-moderate ASD vs severe ASD model.

The horizontal axis denotes video frames across time (5 seconds x 30 frames/second = 150 frames). Left vertical axis is the dendrogram, which shows the sequences of merges or splits that occurred during the agglomerative hierarchical clustering. Right vertical axis denotes the different participants included in the testing dataset. Each joint attention task type shows different patterns of attention weight rise/fall at an individual (patient ID) and diagnostic group (non-ASD vs mild-moderate ASD vs severe ASD) level. Darker shades in the heatmap, representing an increase in attention weight at a certain time step (video frame), correlated with changes in motion or eye-gaze shifting, as confirmed by the Grad-CAM results. Cluster analysis results, as visualized by the dendrograms, reveal that response to joint attention tasks form clusters each consisted of either non-ASD (green), mild-moderate ASD (yellow), or severe ASD (blue) based on heatmap patterns.

Abbreviations: ASD, autism spectrum disorder; IJA, initiation of joint attention; RJA_{low}, response to low joint attention level; RJA_{high}, response to high joint attention level

© 2023 Ko C et al. *JAMA Network Open*.

eTable 1. Compliance Score Metric

Score	Task Type		
	IJA	RJA_{low}	RJA_{high}
1	Participant looks off to space unrelated to the task (no response)	Makes no effort to shift eye gaze (no response)	Makes no effort to shift eye gaze (no response)
2	Makes 1 gaze shift (looks at toy)	Delayed response + short duration of fixed gaze	Delayed response + short duration of fixed gaze
**3**	Makes 1 gaze shift (looks at examiner)	Delayed response + long duration of fixed gaze	Delayed response + long duration of fixed gaze
4	Makes 2 gaze shifts (toy—examiner—toy)	Rapid response + short duration of fixed gaze	Rapid response + short duration of fixed gaze
**5**	Makes 2 gaze shifts (examiner—toy—examiner)	Rapid response + long duration of fixed gaze	Rapid response + long duration of fixed gaze

Definitions:
- Delayed response: ≥1 second delay in response to joint attention (1 second is equivalent to 30 frames)
- Short duration of fixed gaze: less than 3 seconds (where total video duration is 5 seconds)

Scoring guideline:
- Scores of 4 and 5 trump scores of 2 or 3

Abbreviations: IJA, initiation of joint attention; RJA_{low}, response to a low level of joint attention; RJA_{high}, response to a high joint attention level

© 2023 Ko C et al. *JAMA Network Open*.

eTable 2. Performance of Deep Learning System For 3 Joint Attention Types—Training and Validation

ASD vs TD

Task name	Training: No. of videos ASD	Training: No. of videos TD	Training AUROC	Training Accuracy	Training Precision	Training Recall	Validation: No. of videos ASD	Validation: No. of videos TD	Validation AUROC	Validation Accuracy	Validation Precision	Validation Recall
IJA	291.3(±4.5)	378.1(±4.2)	100.0(±0.0)	99.8(±0.3)	99.8(±0.4)	99.7(±0.6)	40.8(±5.5)	46.7(±3.3)	100.0(±0.1)	98.9(±1.7)	98.2(±3.0)	99.5(±1.1)
RJA_{low}	284.3(±6.4)	201.5(±1.1	100.0(±0.1)	99.5(±1.5)	99.4(±2.2)	99.7(±0.6)	39.7(±2.8)	25.3(±1.0)	100.0(±0.1)	99.5(±1.1)	99.5(±1.0)	99.7(±1.2)
RJA_{high}	232.2(±5.7)	157.1(±1.5)	100.0(±0.1)	99.9(±0.2)	99.9(±0.4)	100.0(±0.1)	34.2(±3.0)	20.2(±0.7)	100.0(±0.0)	99.5(±1.1)	100.0(±0.0)	99.1(±1.8)

Non-ASD vs mild-moderate ASD vs severe ASD

Task name	Training: No. of videos Non-ASD	Training: No. of videos Mild-moderate ASD	Training: No. of videos Severe ASD	Training AUROC	Training Accuracy	Training Precision	Training Recall	Validation: No. of videos Non-ASD	Validation: No. of videos Mild-moderate ASD	Validation: No. of videos Severe ASD	Validation AUROC	Validation Accuracy	Validation Precision	Validation Recall
IJA	378.3(±5.1)	85.9(±6.7)	204.5(±8.1)	83.5(±1.2)	86.9(±1.1)	77.9(±1.4)	86.9(±1.1)	46.5(±3.5)	17.6(±3.5)	24.1(±3.6)	85.6(±8.1)	78.6(±4.5)	68.3(±4.4)	78.6(±4.5)
RJA_{low}	201.3(±1.1)	85.6(±3.8)	197.7(±6.9)	85.4(±3.1)	80.2(±5.5)	69.9(±1.6)	80.2(±5.5)	25.5(±1.2)	16.1(±1.4)	24.6(±3.5)	86.0(±7.6)	74.8(±2.1)	60.8(±2.0)	74.8(±2.1)
RJA_{high}	156.6 (±2.0)	68.9(±2.5)	165.1(±6.4)	84.0(±1.5)	82.2(±1.0)	69.8(±1.2)	82.2(±1.0)	20.7(±1.1)	12.1(±1.9)	20.3(±3.0)	85.2(±6.3)	77.2(±3.2)	63.1(±4.1)	77.2(±3.2)

* The mean and standard deviation were calculated from all the models based on 10-fold cross validation.
Abbreviations: ASD, autism spectrum disorder; AUROC, area under the receiver operating characteristic curve; IJA, initiation of joint attention; RJA_{low}, response to low joint attention level; RJA_{high}, response to high joint attention level; TD, typical development

© 2023 Ko C et al. *JAMA Network Open*.

eReferences

1. American Psychiatric Association. Diagnostic and Statistical Manual of Mental Disorders. American Psychiatric Association; 2013. doi:10.1176/appi.books.9780890425596
2. Hedley D, Nevill R, Uljarević M, Butter E, Mulick JA. ADOS-2 Toddler and Module 1 standardized severity scores as used by community practitioners. Res Autism Spectr Disord. 2016;32:84-95. doi:10.1016/j.rasd.2016.09.005
3. Luyster R, Gotham K, Guthrie W, et al. The autism diagnostic observation schedule - Toddler module: A new module of a standardized diagnostic measure for autism spectrum disorders. *J Autism Dev Disord*. 2009;39(9):1305-1320. doi:10.1007/s10803-009-0746-z
4. Hus V, Gotham K, Lord C. Standardizing ADOS domain scores: Separating severity of social affect and restricted and repetitive behaviors. J Autism Dev Disord. 2014;44(10):2400-2412. doi:10.1007/s10803-012-1719-1
5. Chlebowski C, Green JA, Barton ML, Fein D. Using the childhood autism rating scale to diagnose autism spectrum disorders. J Autism Dev Disord. 2010;40(7):787-799. doi:10.1007/s10803-009-0926-x
6. Park HS, Yi SY, Yoon SA, Hong SB. Comparison of the Autism Diagnostic Observation Schedule and Childhood Autism Rating Scale in the Diagnosis of Autism Spectrum Disorder: A Preliminary Study. Journal of the Korean Academy of Child and Adolescent Psychiatry. 2018;29(4):172-177. doi:10.5765/jkacap.180015
7. Mundy P, Delgado C, Block J, et al. *DRAFT A Manual for the EARLY SOCIAL COMMUNICATION SCALES (ESCS)*. 2003. http://edscholars.ucdavis.edu/vrlab/home
8. He K, Zhang X, Ren S, Sun J. Deep Residual Learning for Image Recognition. In: *2016 IEEE Conference on Computer Vision and Pattern Recognition (CVPR)*. IEEE; 2016:770-778. doi:10.1109/CVPR.2016.90
9. Peng P, Wang J. How to fine-tune deep neural networks in few-shot learning? Published online November 30, 2020.
10. Zhang D, Li J, Shan Z. Implementation of Dlib Deep Learning Face Recognition Technology. In: *2020 International Conference on Robots & Intelligent System (ICRIS)*. IEEE; 2020:88-91. doi:10.1109/ICRIS52159.2020.00030

© 2023 Ko C et al. *JAMA Network Open*.
