# Supplementary material for: Development and Validation of a Joint Attention–Based Deep Learning System for Detection and Symptom Severity Assessment of Autism Spectrum Disorder
Source: JAMA Netw Open. 2023 May 25;6(5):e2315174. doi: 10.1001/jamanetworkopen.2023.15174 (PMC10214037; doi:10.1001/jamanetworkopen.2023.15174)
Supplement: Supplement 2. — Data Sharing Statement [file jamanetwopen-e2315174-s002.pdf]

Data Sharing Statement

Ko. Development and Validation of a Joint Attention–Based Deep Learning System for Detection and Symptom Severity Assessment of Autism Spectrum Disorder. *JAMA Netw Open*. Published May 25, 2023. doi:10.1001/jamanetworkopen.2023.15174

Data
Data available: No

Additional Information
Explanation for why data not available: It can be discussed through contact with the corresponding author(Yu Rang Park).
